# One-Pot Synthesis of P(*O*)-N Containing Compounds Using *N*-Chlorosuccinimide and Their Influence in Thermal Decomposition of PU Foams

**DOI:** 10.3390/polym10070740

**Published:** 2018-07-05

**Authors:** Khalifah A. Salmeia, Florence Flaig, Daniel Rentsch, Sabyasachi Gaan

**Affiliations:** 1 Additives and Chemistry Group, Advanced Fibers, Empa—Swiss Federal Laboratories for Materials Science and Technology, Lerchenfeldstrasse 5, 9014 St. Gallen, Switzerland; florence.flaig@etu.unistra.fr; 2 Laboratory for Functional Polymers, Empa—Swiss Federal Laboratories for Materials Science and Technology, Überlandstrasse 129, 8600 Dübendorf, Switzerland; daniel.rentsch@empa.ch

**Keywords:** chlorination reaction, phosphoramidates, phosphonamidates, thermal decomposition, polyurethane foams

## Abstract

Synthesis of intermediate containing P(*O*)-Cl bonds is the key to converting P(*O*)-H bonds to P(*O*)-N. In this work we have performed chlorination reactions of different *H*-phosphinates and *H*-phosphonates using *N*-chlorosuccinimide as an environmentally-benign chlorinating agent. The chlorination reaction showed high yield and high selectivity for transformation of P(*O*)-H bonds into P(*O*)-Cl analogues, resulting in an easily separable succinimide as the by-product. Using a one-pot synthesis methodology, we have synthesized a series of P(*O*)-N containing derivatives whose synthesis was found to be dependent on the reaction solvents and the starting materials. The synthesized P(*O*)-N compounds were incorporated in flexible polyurethane foam (FPUF) and screened for their influence in thermal decomposition of FPUFs using thermogravimetric analysis (TGA) and a microscale combustion calorimeter (MCC). All solid P(*O*)-N compounds influenced the first-stage decomposition of FPUFs, which resulted in an accelerated decomposition or temporary stabilization of this stage. However, the liquid P(*O*)-N derivatives volatilize at an earlier stage and could be active in the gas phase. In addition, they also work in condensed phase via acid catalyzed decomposition for FPUFs.

## 1. Introduction

Over the past century, the use of organophosphorus compounds has been extensively explored due to their versatility [1,2]. This exploration has paved the way to develop new synthetic methodologies and further the design of new organophosphorus compounds [3,4].

Among the latter, the P(*O*)-N containing organophosphorus compounds, namely phosphoramidates and phosphonamidates, have received a great deal of attention owing to their diverse applications. They have been used for treating and controlling various diseases, making them useful candidates for pharmaceutical products [5,6]. Some of them have shown to be excellent flame retardants on polyurethane [7,8,9,10,11], polycarbonates [12,13], polyamide 66 [14], epoxy resins [15,16,17], and cellulose-based fabrics [18,19,20]. Because of this wide range of applications, production of P(*O*)-N containing organophosphorus compounds has gained commercial importance. For the commercial exploitation of such compounds, industrially feasible synthetic strategies need to be developed. Since 1945, Atherton—Todd reaction has been widely employed as a synthetic approach to convert *H*-phosphonates or *H*-phosphinates to phosphoramidates and phosphonamidates, respectively, using CCl_4_ and amines. However, despite the ease of handling of Atherton–Todd reaction and its simplicity for possible industrial upscaling, it has found no commercial success [9] as CCl_4_ is a carcinogen and depletes the ozone [21,22,23]. Thus, alternative environmentally-friendly synthetic strategies for conversion of P(*O*)-H bonds to P(*O*)-N bonds are in high demand. New synthetic approaches have been explored in recent years which focus on benign synthetic principles. The conventional synthetic route for producing phosphoramidates and phosphonamidates involves the reaction of amines with the corresponding phosphorochloridate and phosphonochloridate intermediates, respectively [12,24,25,26]. The P(*O*)-halogen bond plays a crucial role as an intermediate in two-step/one-pot synthetic strategies. In such cases, transformation of P(*O*)-H bonds into P(*O*)-halogen were carried out using equimolar quantities or more of various halogenating agents, for example, tert-butyl hypochlorite [27], CuCl_2_ [28], SO_2_Cl_2_ [29], trichloroisocyanuric acid [29], *N*-bromosuccinimide [30,31], and Cl_2_ [32,33,34]. Michaelis—Arbuzov rearrangement reaction has also been investigated for reaction of trialkyl phosphite with Cl_2_ to produce the corresponding P(*O*)-Cl bond [35]. Moreover, synthesis of phosphoramidates via one-pot reaction was studied using catalytic amounts of Cu catalysts [36,37] or I_2_ [38,39] via an oxidative coupling method. Many synthetic challenges such as producing non-desirable by-products, exothermic nature, use of expensive raw materials, and relatively moderate reaction yield were encountered when applying these methods. New synthetic approaches are also reported to synthesize phosphoramidates via Staudinger-type [40,41] or Staudinger-phosphite [42,43] reactions. The latter two methods involve the reaction of trialkyl phosphite with alkyl azides to produce phosphorimidates, which subsequently undergo rearrangement to their corresponding phosphoramidates. Metal-catalyzed C-H bond amidation is also reported in synthesis of phosphoramidates using phosphorylazides [44,45,46,47]. The only drawback of the latter two methods is the use of hazardous organic azides in the synthesis. Recently, green synthetic methodology was reported to synthesize phosphoramidates from the reaction of *H*-phosphonates with amines using an organic dye as a photocatalyst. However, this synthetic strategy was only suitable for diethyl phosphite compared to other *H*-phosphonates [48].

The overall goal of this work is to synthesize a series of phosphoramidates and phosphonamidates substances via a one-pot synthesis, and to investigate their influence in thermal decomposition of FPUF. Our synthetic strategy involves the use of *N*-chlorosuccinimide (NCS) as a versatile and simple chlorinating agent. It is a well-known chlorinating agent; however, just few reports exist about its use in producing the organophosphorus intermediate containing P(*O*)-Cl bonds [49,50,51]. Unlike most other chlorinating agents, a key benefit of using NCS is it results in the formation of the benign by-product succinimide. The use of NCS in such reactions can be convenient to develop green and effective low-cost synthetic routes for industrial exploitation. As phosphoramidates and phosphonamidates are important as flame retardant additives, the influence of all synthesized P(*O*)-N substances on thermal decomposition FPUF was evaluated using TGA and MCC. This was estimated by incorporating them in flexible polyurethane foams (FPUF) and performing thermal characterisations.

## 2. Materials and Methods

### 2.1. Materials

Diethyl phosphite (DEP; 98% purity) and diphenyl phosphite (DPP; ≤15% phenol impurities) were purchased from Sigma-Aldrich (Buchs, Switzerland) and used as received. 9,10-Dihydro-9-oxa-10-phosphaphenanthrene 10-oxide (DOPO; >97% purity) was purchased from Metadynea Austria GmbH (Krems, Austria), recrystallized from Toluene, and dried under vacuum at 80 °C prior to use. *N*-chlorosuccinimide (98% purity) was purchased from Sigma-Aldrich, recrystallized from water, and dried in a vacuum oven. Propyl amine (PA; 98% purity), benzyl amine (BA; ReagentPlus^®^, 99% purity), diethyl amine (DA; ≥99% purity), allyl amine (AA; ≥99% purity), and ethylene diamine (EDA; for synthesis) were purchased from Sigma-Aldrich, dried using standard methods, and stored under N_2_ [52]. Materials used in the production of flexible PU foams: PO 56 (propylene oxide polyol with a hydroxyl value of 56 mg KOH/g and molecular weight of 3000 g/moL) from Covestro, formerly Bayer MaterialScience AG (Leverkusen, Germany), KOSMOS EF (catalyst of tin ricinoleate) from Evonik Industries AG (Essen, Germany), Tegoamin ZE1 (amine catalyst, blowing catalyst) from Evonik Industries AG. STABI AKTIV (silicone surfactant) from Momentive Performance Materials GmbH (Leverkusen Germany) were supplied by FoamPartner AG (Wolfhausen, Switzerland). T80 (technical grade; a mixture of 2,4-toluene diisocyanate and 2,6-toluene diisocyanate isomers with ratio 80:20, respectively) was purchased from Sigma-Aldrich, Switzerland.

### 2.2. Thermal and Fire Characterization, ICP-OES and NMR

Heat release rates (HRRs) of FPUFs were determined using a microscale combustion calorimeter (MCC) (Fire Testing Technology Instrument UK) following ASTM D7309. Each sample (about 2 mg) was exposed to a heating rate of 1 °C·s^−1^ from 100 °C to 700 °C in the pyrolysis zone and at a combustion temperature of 900 °C. The thermogravimetric analysis (TGA) of FPUFs was performed on a NETZSCH TG 209 F1 instrument (NETZSCH-Gerätebau GmbH, Selb, Germany). Tests were conducted on 2–5 mg sample under N_2_ atmosphere and under air at a heating rate of 10 °C/min from 25 °C to 800 °C. The TGA curves represent the average of two measurements. Differential scanning calorimetry (DSC) analyses were performed on DSC 214 Polyma instrument (NETZSCH-Gerätebau GmbH, Selb, Germany). All experiments were conducted at a controlled heating rate of 10 °C/min under nitrogen flow of 50 mL/min. Phosphorus-contents of FPUFs samples and P(*O*)-N derivatives were determined using PerkinElmer OPTIMA 3000 ICP-OES (PerkinElmer AG, Rotkreuz, Switzerland). The parameters of the NMR spectroscopy are summarized in the Supporting Information and fully assigned ^1^H, ^13^C and ^31^P NMR spectra are shown in Figures S1–S12.

### 2.3. Synthesis

#### 2.3.1. Synthesis of Phosphonamidates (General Procedure)

To a stirred solution of DOPO (5.0 g, 23 mmoL) in dichloromethane (50 mL), NCS (3.40 g, 25 mmoL) was gradually added under nitrogen atmosphere at 0 °C. The reaction mixture was then allowed to warm to ambient temperature and stirred for additional 2 h. Triethylamine (3.87 mL, 28 mmoL) and the corresponding amine (28 mmoL) were then added drop-wise to the reaction mixture at 0 °C. The reaction mixture was then stirred at room temperature overnight. Volatiles were then completely removed and the solid was stirred in water and then separated by filtration (except 6-(diethylamino)dibenzo[c,e][1,2]oxaphosphinine 6-oxide (DA-DOPO)). All compounds were fully characterized and found identical to the published data [9,29,53].

#### 2.3.2. Synthesis of Phosphoramidates (General Procedure)

● Synthesis of DPP based phosphoramidates:

To a stirred solution of diphenyl phosphite (4.09 mL, 21 mmoL) in dichloromethane (50 mL), NCS (3.14 g, 24 mmoL) was gradually added under nitrogen atmosphere at 0 °C. The reaction mixture was then allowed to warm to ambient temperature and stirred for additional 2 h. Triethylamine (3.28 mL, 24 mmoL) and the corresponding amine (24 mmoL) in dichloromethane (10 mL) were then added drop-wise at 0 °C. The reaction mixture was then stirred at ambient temperature overnight. Volatiles were then completely removed under vacuum and the residue was stirred in diethyl ether and filtered. The volatiles were then completely removed from the filtrate under vacuum. After purifications, all compounds were fully characterized and found identical to the published data [8,54,55,56].

● Synthesis of DEP based phosphoramidates:

To a stirred solution of diethyl phosphite (4.66 mL, 36 mmoL) in dichloromethane (50 mL), NCS (5.32 g, 40 mmoL) was gradually added under nitrogen atmosphere at 0 °C. The reaction mixture was then allowed to warm to ambient temperature and stirred for additional 2 h. Triethylamine (5.56 mL, 40 mmoL,) and the corresponding amine (40 mmoL) were then drop-wise added at 0 °C. The reaction mixture was stirred at ambient temperature overnight. Volatiles were then completely removed and the oily residue was stirred in diethyl ether (except diethyl benzylphosphoramidate (BA-DEP) and tetraethyl ethane-1,2-diylbis(phosphoramidate) (EDA-DEP)) and filtered. The volatiles were then removed from the filtrate under vacuum. After purification, all the compounds were fully characterized and found identical to the published data [19].

### 2.4. Preparation of FPUF

FPUFs were manufactured by incorporating suitable concentration of synthesized phosphonamidates and phosphoramidates compounds so as to obtain 2 wt % phosphorus in the resulting foams [8]. The required quantities of the polyol (100 parts), phosphorus compounds (parts based on wt % of P-content), water (1.6 parts), Tegoamine ZE1 (0.4 parts), STABI AKTIV (0.5 parts), and KOSMOS EF (0.45 parts) were mixed in a plastic cup with digital homogenizer Ultra Turrax T18 from IKA at speed of 9000 rpm for 90 s. Subsequently, T80 (26.20 parts) was added during continuous stirring of the reaction components. The mixture was then stirred for a further 6 s and immediately transferred into a container to rise freely. The samples were then cured at 80 °C for 1 h and allowed to cool down to ambient temperatures. All prepared FPUFs were conditioned for 48 h at 25 °C and 50% of relative humidity prior to further analysis.

## 3. Results and Discussion

### 3.1. Synthesis of the P(O)-N Compounds

**Scheme 1:**
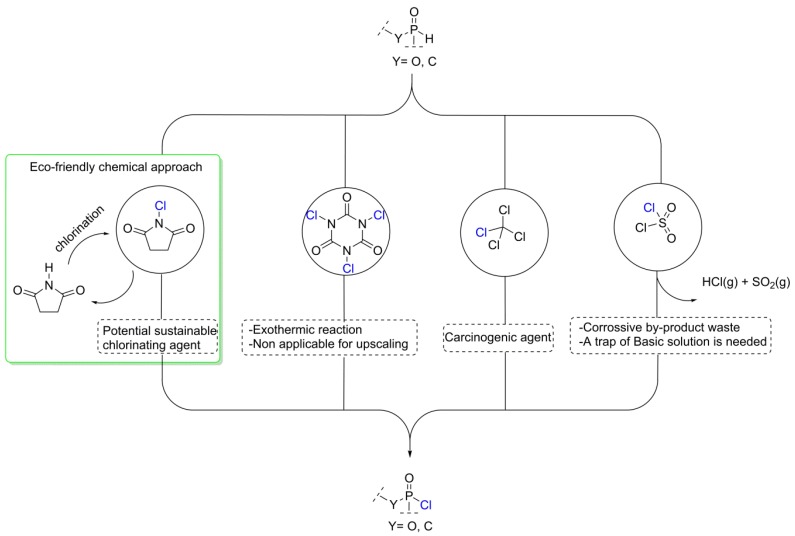
Comparison of the chemical transformation reactions of P(*O*)-H into P(*O*)-Cl bonds using different chlorinating agents.

The advantage of the transformation reaction of P(*O*)-H bonds into its corresponding P(*O*)-N bonds via intermediate chlorinating step with NCS is the production of succinimide as a benign by-product. The latter can be recovered to regenerate *N*-chlorosuccinimde, mitigating any potential chemical by-product wastes [57,58]. Thus, the use of NCS can be considered as an alternative sustainable green chlorination agent compared to CCl_4_, SO_2_Cl_2_, and trichlorocyanuric acid (Scheme 1) [29].

The transformation reaction of P(*O*)-H bonds into P(*O*)-Cl (of respective diethyl phosphorochloridate, diphenyl phosphorochloridate, and 6-chlorodibenzo[c,e][1,2]oxaphosphinine 6-oxide reaction) was confirmed by ^31^P NMR spectroscopy of the reaction mixture after the first step. All chloro-derivatives were also isolated and their chemical and physical properties were found to be consistent with their reported data [59,60]. The initial reaction screening showed that the transformation of the P(*O*)-H bond into its corresponding P(*O*)-Cl is affected by the impurities present in the starting material (Table 1). For instance, in the synthesis of diphenyl phosphorochloridate (Table 1, entry 2) we observed the formation of triphenyl phosphate, which is due to the presence of residual phenol in diphenyl phosphite.

The transformation reaction of P(*O*)-H bonds into P(*O*)-Cl bonds using NCS was optimized using PA-DOPO as a model derivative. Thus the synthesis of PA-DOPO was optimized using the general synthetic procedure, using different solvent/base pairs. The results are summarized in Table 2.

**Figure 1:**
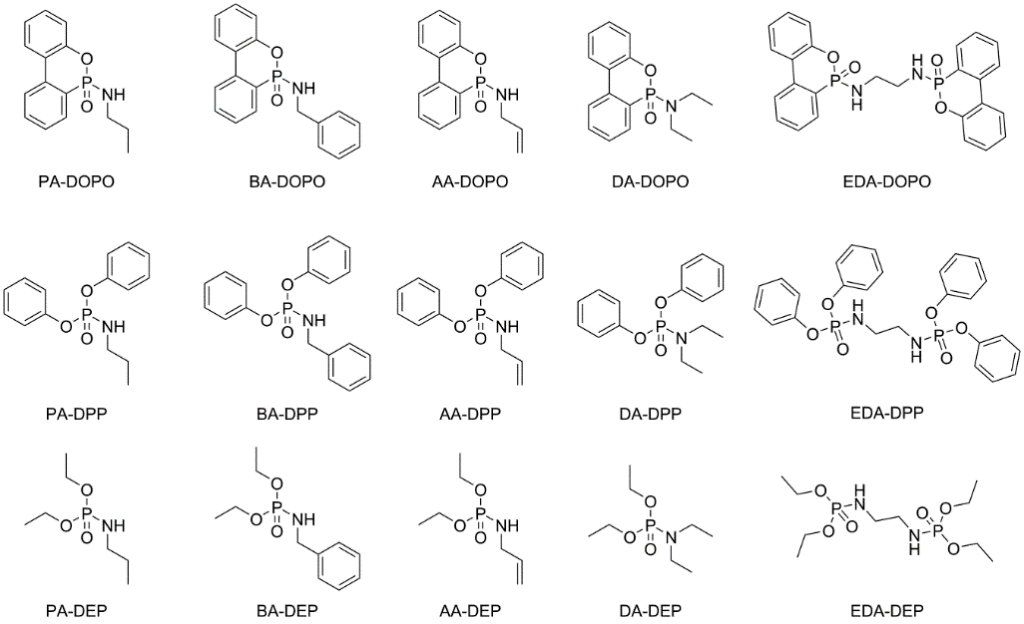
Chemical structures of phosphonamidates and phosphoramidates derivatives.

It was observed that the reaction mixtures in chloroform, toluene, and acetonitrile (Table 2, Entries 1–3) turned dark brown upon addition of PA. After removal of the reaction volatiles, dark residue was obtained which required further purification steps to afford a clean PA-DOPO. On the other hand, a white residue was obtained when dichloromethane and triethylamine were used as a solvent and an organic base, respectively (Table 2, Entry 4). On washing the white residue with water, a clean PA-DOPO was obtained without any further purification procedure. By changing the organic base to inorganic base (Table 2, Entry 5), a relatively lower yield of PA-DOPO was obtained which can be attributed to the poor solubility of the inorganic base in organic solvents. Subsequently, all phosphonamidates and phosphoramidates (Figure 1) were synthesized based on the optimized synthetic approach (Table 2, Entry 4).

The phosphonamidate derivatives of DOPO compound are water insoluble, which simplifies their cleaning procedure. They are purified by washing away the crude products with water to remove the triethylamine hydrochloride salt and the succinimide, affording clean derivatives in relatively moderate to high yields (Table 3, Entry 1). Following the same cleaning methodology, the phosphoramidate derivatives of DPP were cleaned by washing the crude products with water. However, both PA-DPP and DA-DPP derivatives (Table 3, Entry 2) required a modified cleaning procedure using column chromatography for satisfactory purity. This procedure was found to reduce the overall reaction yield. To overcome the low yield of PA-DPP and DA-DPP, attempts were made to isolate diphenyl phosphorochloridate from succinimide by vacuum distillation to avoid the side reactions. As the boiling point of diphenyl phosphorochloridate is relatively high, the succinimide impurity could separate and become condensed in the distillation apparatus. However, the phosphoramidate derivatives of DEP are water soluble and cannot be purified by washing them with water. Accordingly, these derivatives were isolated from the reaction mixture either by dissolving them in diethyl ether or THF or by vacuum distillation (Table 3, Entry 3). It is noteworthy to mention that due to the low boiling point of diethyl phosphorochloridate at low pressure, it can be quantitatively isolated from the reaction mixture in high purity via distillation. It can be then stored and subsequently used for further chemical reactions.

### 3.2. Proposed Chlorination Mechanism

**Scheme 2:**
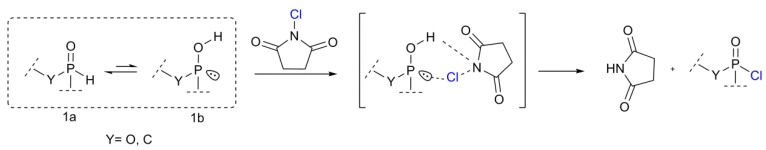
Proposed mechanism of the chlorination reaction using NCS.

The *H*-phosphinate (DOPO) and *H*-phosphonates (DEP and DPP) are considered tetra-coordinated P(III) compounds [2]. As these compounds resemble the chemical structure of the tetra-coordinated of P(V) compounds (phosphonates), the P-center is predicted to be an electrophilic center. The electrophilic property can be modified to be a nucleophilic due to their tautomeric equilibrium in a solution between the tetra-coordinated (1a) and tri-coordinated (1b) forms (Scheme 2).

The Cl atom has a positive charge due to the influence of the electron-withdrawing ability of an oxygen atom on NCS, making it susceptible to attack by a nucleophilic center [61], namely the tri-coordinated P-atom; this produces the succinimide and the corresponding chlorophosphate derivative. Based on a previous report with regards to chlorination of secondary amine using NCS, the chlorination reaction occurs in stoichiometric ratio and the exchange of chlorine for hydrogen happens directly (Scheme 2) [62]. The kinetic studies of the chlorination reaction using NCS are reported to take place via first order with respect to NCS [63,64].

### 3.3. Thermal Analysis

The thermogravimetric analysis is viewed as an assisted tool to investigate the possible effect of the incorporated flame retardant additives on the polymer matrix [65]. Therefore, the influence of the synthezised phosphorus compounds on FPUF decomposition was investigated in a non-oxidative environment and relevant data are summarized in Table 4.

**Figure 2:**
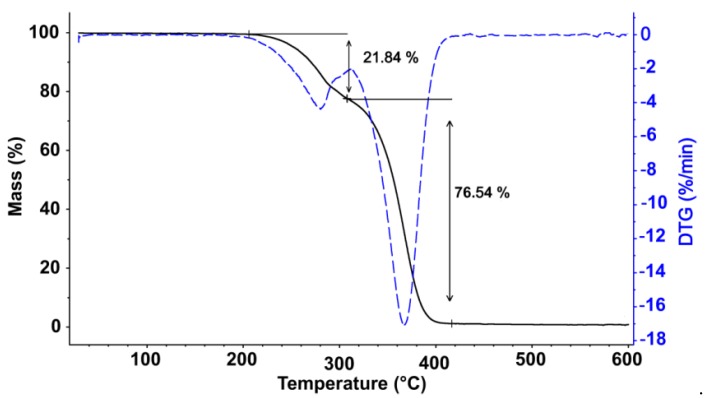
TGA (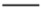) and derivative thermogravimetry (DTG) (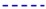) curves for pure FPUF under N_2_ atmosphere.

The thermogravimetric analysis of virgin FPUF (Figure 2) showed a two-stage decomposition process from 200 °C to 420 °C, which is consistent with the literature data [7,66].

**Figure 3:**
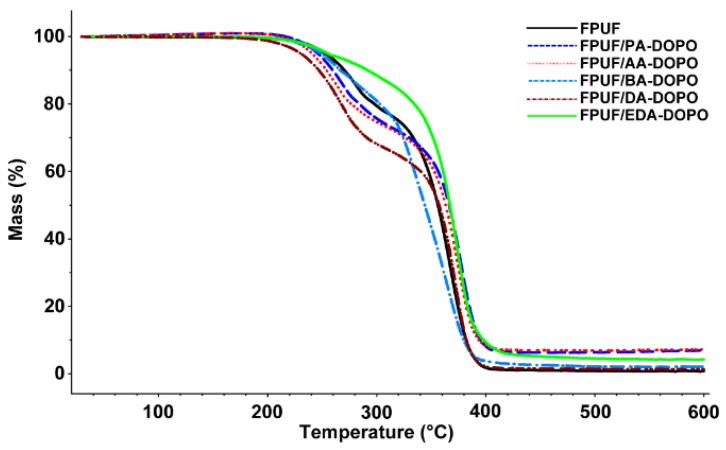
TGA curves of treated FPUF with flame retardant derivatives of DOPO compound in comparison to pure FPUF under N_2_ atmosphere.

The first stage decomposition occurs between 200 °C and 320 °C with around 22% mass loss (Figure 2), which is attributed to decomposition of the urethane linkages to produce isocyanate and its derivatives [67]. The second stage decomposition corresponds to the decomposition of the polyether main chain at about 320 °C till 420 °C, with around 77% mass loss [68]. After which, a gradual mass loss takes place with a negligible char residue (Table 4). On the other hand, the addition of flame retardants to FPUF alters the decomposition profile of the FPUF either via volatilization of the phosphorus compounds or their decomposition products during the first stage decomposition of FPUFs. For example, the thermal decomposition of FPUF containing PA-DOPO, AA-DOPO, and DA-DOPO showed an accelerated decomposition in the first-stage (Figure 3), which may be attributed to the catalytic influence on the decomposition of the urethane bond by the degradation products of phosphorus additives.

**Figure 4:**
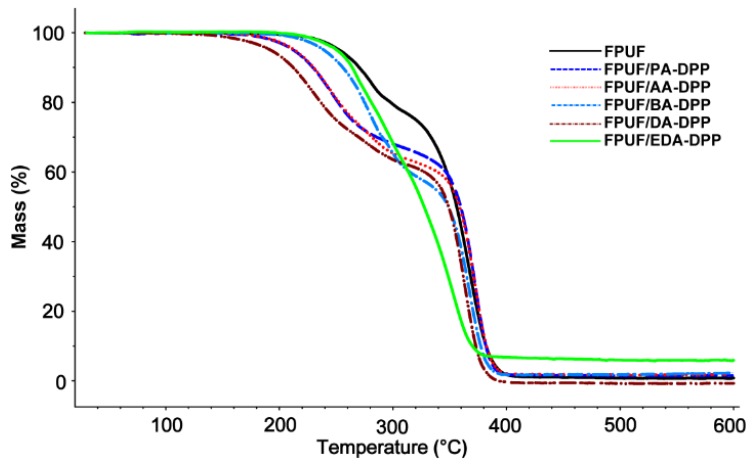
TGA curves of treated FPUF with flame retardant derivatives of DPP compound in comparison to pure FPUF under N_2_ atmosphere.

It indicates that these phosphorus additives can act in the gas phase at a relatively low temperature [9]. After the first-stage decomposition, the second-stage decomposition profile of the FPUF contained DA-DOPO is identical to the virgin FPUR, resulting in a complete decomposition and no residue (Table 4). On the contrary, both PA-DOPO and AA-DOPO derivatives influence the second-stage decomposition by decreasing the degradation rate of the polyether main chain, affording a relative increase in the char residue of the treated FPUF. This clearly indicates the possibility of a condensed phase action of the additives. BA-DOPO did now show any influence on the first-stage decomposition of FPUF, but accelerated the rate of decomposition of the second stage, leading to complete decomposition of the FPUF. However, EDA-DOPO containing FPUF showed an increase in temperature for both first-stage and second-stage decomposition, which is attributed to the high-thermal stability of the EDA-DOPO additive and its possible condensed phase action [9]. One can speculate that EDA-DOPO is relatively stable and melts at a temperature (266 °C) which matches well with the first-stage decomposition of FPUF. At this stage, the molten EDA could react with isocyanate released to form allophanate structures and prevents its volatilization resulting in increased residue. The FPUFs containing DPP based phosphoramidates showed similar decomposition profiles (Figure 4).

**Figure 5:**
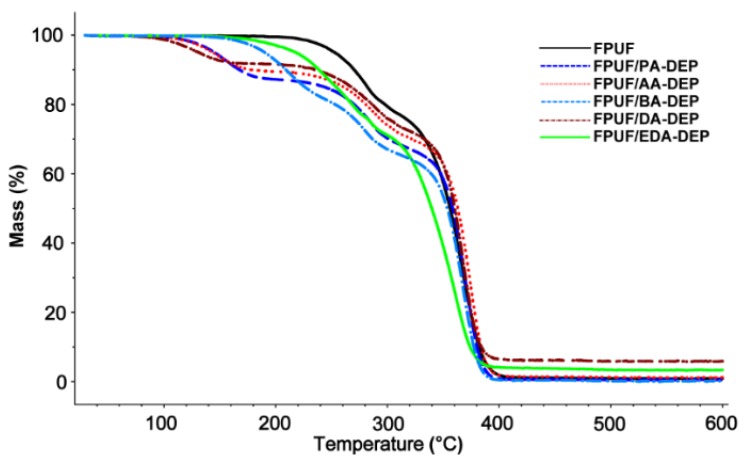
TGA curves of treated FPUF with flame retardant derivatives of DEP compound in comparison to pure FPUF under N_2_ atmosphere.

The first-stage decomposition of FPUF was accelerated in the presence of PA-DPP, AA-DPP, BA-DPP, and DA-DPP. This influence can be attributed to the acid-catalyzed depolymerization of the urethane bond from the acidic by-products formed from the decomposition of the phosphorus compounds [8]. This observation clearly indicates condensed phase action of these phosphorus compounds. On the contrary, the EDA-DPP containing FPUF exhibited only one stage decomposition process, with a slight increase in the residue. This observation indicates that EDA-DPP can also act in the condensed phase. The thermal decomposition FPUF containing phosphoramidate additives of DEP showed clear three-stage decomposition, except EDA-DEP containing FPUF (Figure 5).

Mass loss around 130–180 °C can be attributed to the volatilization of PA-DEP, AA-DEP, BA-DEP, and DA-DEP from the FPUF matrix (Figure S13a–d). The volatilized additives can thus act in the gas phase. The remaining trapped additives in the FPUF matrix accelerate the first-stage decomposition, possibly due to the formation of non-volatile acidic species formed from their thermal decomposition [7,8,9,69]. PA-DEP, AA-DEP, BA-DEP, and DA-DEP did not influence the second stage thermal decomposition corresponding to polyether main chains. However, EDA-DEP showed an accelerated degradation rate of the second-stage decomposition of the FPUF, but with a slight increase of the residual mass. In light of this, the phosphoramidate derivatives of DEP act mainly in the gas phase with limited action in the condensed phase for both DA-DEP and EDA-DEP.

### 3.4. Small Scale Fire Test with Micro Scale Combustion Calorimeter

The objective of this work was to screen series of analogues P(*O*)-N containing compounds to gain preliminary info on their flame retardant behavior and efficacy. MCC is a useful instrument to evaluate the flame retardant performance in milligram scale. Although the milligram scale analysis may not represent the fire performance in large scale, it can, however, provide valuable information regarding mode of action of flame retardant additives [65]. To investigate the efficacy of synthesized phosphorus compounds, FPUFs were manufactured with suitable concentration of phosphorus compounds with P-content of around 2 wt %. The MCC data of the pure FPUF and the flame retarded FPUFs were summarized in Table 5.

**Figure 6:**
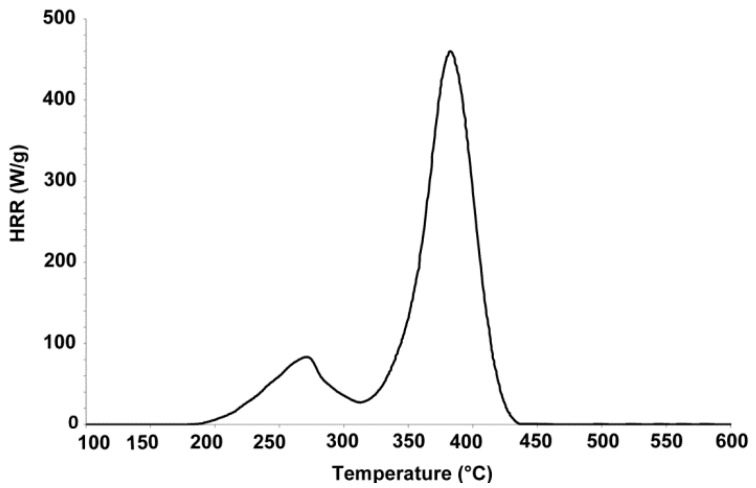
Heat-release rate (HRR) curve of pure FPUF.

The heat release rate (HRR) of the pure FPUF as a function of temperature (Figure 6) showed a two-stage heat release, which is consistent with the TGA data. The first stage of HRR between 300 °C to 320 °C represents the heat release due to thermal decomposition of the urethane bond, affording the volatile isocyanate products. However, the second-stage HRR (320 °C–450 °C) corresponds to the thermal decomposition of the polyether main chain.

It is noteworthy to mention that reduced HRR is evidence of reduced fuel production. In MCC, reduced heat release rate and increased char formation is a clear sign of the condensed phase mode of action. The HRR profile of FPUF/EDA-DOPO (Figure S14a) compared to the virgin FPUF shows a significant decrease of the HRR in the first-stage decomposition of the FPUF, which can be attributed to its positive influence in retarding the 1st stage decomposition of FPUF. However, the THR of flame retarded FPUF compared to the virgin FPUF (Table 5, Figure S15) did not show any significant decrease, indicating that phosphoramidates and phosphonamidates additives act primarily during the gas phase. On the other hand, BA-DOPO and EDA-DPP showed a decrease in HRC (Figure S17), revealing that the pyrolysis of these two additives interfere with the second-stage pyrolysis of FPUF, thus reducing the degradation rate of the main chain polyether, allowing these two additives to act somehow in the condensed phase. However, the absence of the char residue clearly shows that these additives cannot create thermally stable residue, and thus may primarily work in the gas phase.

## 4. Conclusions

The synthesis of various P(*O*)-N containing phosphoramidates and phosphonamidates from their corresponding *H*-Phosphonates and *H*-Phosphinates, respectively, was investigated using one pot two-step process with *N*-chlorosuccinimide as a versatile and environmentally friendly chlorinating agent. *N*-chlorosuccinimide as a chlorinating agent showed high selectivity and high yield, affording succinimide as a benign by-product. Accordingly, this synthetic methodology can be considered as an eco-friendly approach. However, more studies are needed to evaluate the economic and commercial benefits of this method. In this study, we mainly focused on in situ synthesis of P(*O*)-Cl to investigate the feasibility for production of corresponding P(*O*)-N containing derivatives and evaluating their influence in the thermal decomposition and possible mode of action in FPUF. In this work we used amines such as propylamine, allylamine, benzylamine, diethylamine, and ethylenediamine and converted them in situ to their corresponding P(*O*)-N derivatives. The final products were obtained in high yields and high purity even in the presence of succinimide, which can be removed by using a suitable purification procedure. Preliminary investigations regarding their influence in thermal decomposition of FPUFs was evaluated using TGA and MCC and their possible mode of flame retardant action was proposed. Comparing the TGA data of pure FPUF with those containing phosphoramidate or phosphonamidate showed that they have an influence on the decomposition pathways of the FPUF, which is generally limited to the first-stage decomposition of the FPUF, where a catalyzed depolymerization of the urethane linkages takes place. All solid P(*O*)-N compounds influenced the first stage decomposition of FPUFs which resulted in an accelerated decomposition or temporary stabilization of this stage. However, the liquid P(*O*)-N derivatives volatilize at an earlier stage and could be active in the gas phase. In addition, they also work in condensed phase via acid-catalyzed decomposition for FPUFs. THR, HRC, and PHRR data obtained in MCC experiments further highlighted the potential gas phase flame inhibition of these P(*O*)-N substances, with a limited condensed phase mode of action. The aromatic-containing FR additives were noticed to act in the condensed phase, but resulted in negligible char residue at 800 °C. This is attributed to the complete thermal decomposition of FPUF and absence of any dehydration process which prevents formation of the voluminous char. In general, use of *N*-chlorosuccinimide as a chlorinating agent is a relatively simple and clean synthetic approach to produce P(*O*)-N containing phosphorus derivatives. Therefore, future work will involve synthesis of phosphates, phosphonates, and phosphinates by using this synthetic approach. In addition detailed fire evaluation of all synthesized phosphorus compounds and a comprehensive mechanistic study will also be carried out and correlated to the data obtained in this research.

## Figures and Tables

**Table 1:** Chlorination reaction of different organophosphorus compounds using *N*-chlorosuccinimide (NCS).

Entry	Starting Material	Product ^(a)^	Yield (%) ^(b)^	^31^P NMR (ppm) ^(c)^
1	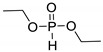	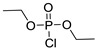	100	4.1
2 ^(d)^	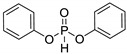	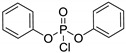	95	−5.1
3 ^(e)^	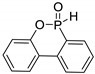	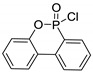	98	19.7

^(a)^ The starting material (231 mmoL) was charged in three-neck round bottom flask under N_2_ atmosphere. Toluene (30 mL) was then added and the reaction mixture was cooled to 0 °C. NCS (254 mmoL) was then added in small portions and the reaction was then allowed to warm up to ambient temperature and stirred for 2 h. The precipitate was then removed by filtration using Schlenk-frit and the volatiles were removed under vacuum, affording the desired product; ^(b)^ The resulting products were analyzed by GC-MS and NMR spectroscopy; ^(c) 31^P NMR spectra were recorded in CDCl_3_; ^(d)^ Diphenyl phosphite contained ≤15% phenol and used as received; ^(e)^ DOPO was recrystallized from toluene and dried in vacuum at 80 °C prior to use.

**Table 2:**
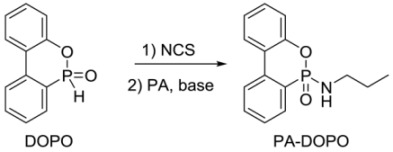
The optimization of the chlorination reaction using PA-DOPO as a model derivative.

Entry	Solvent	Base	Yield (%)
1	chloroform	triethylamine	62
2	toluene	triethylamine	48
3	acetonitrile	triethylamine	55
4	dichloromethane	triethylamine	90
5	dichloromethane	K_2_CO_3_	60

**Table 3:** Physical properties of the phosphonamidates and phosphoramidates.

Entry	Substances	Appearance	mp ^(a)^ (°C)/bp ^(b)^ (°C)	Yield (%)
	PA-DOPO	White solid	131 ^(a)^	90
	AA-DOPO	White solid	103 ^(a)^	91
1	BA-DOPO	Off-white solid	159 ^(a)^	98
	DA-DOPO	Red solid	114 ^(a)^	59
	EDA-DOPO	White solid	266 ^(a)^	75
	PA-DPP	Off-white solid	55 ^(a)^	68
	AA-DPP	White solid	55 ^(a)^	83
2	BA-DPP	Off-white solid	106 ^(a)^	87
	DA-DPP	White solid	55 ^(a)^	63
	EDA-DPP	White solid	134 ^(a)^	85
	PA-DEP	Colorless oil	82–85 ^(b)^ at 0.13 mbar	81
	AA-DEP	Pale yellow oil	ND	68
3	BA-DEP	Pale Yellow oil	ND	94
	DA-DEP	Pale yellow oil	55–56 ^(b)^ at 0.29 mbar	74
	EDA-DEP	Off-white solid	83	95

^(a)^ mp (°C); ^(b)^ bp (°C).

**Table 4:** TGA data of flame retarded FPUF.

Sampless	Stage 1		Stage 2		Stage 3		Char Residue at 600 °C (wt %) ± 0.5
	*T*_onset_ (°C)	*T*_max_^(a)^ (°C)	*T*_onset_ (°C)	*T*_max_^(a)^ (°C)	*T*_onset_ (°C)	*T*_max_^(a)^ (°C)	
FPUF	262	282	346	367	---	---	1
FPUF/PA-DOPO	249	267	354	371	---	---	7
FPUF/AA-DOPO	238	266	357	374	---	---	7
FPUF/BA-DOPO	251	320	262	365	---	---	2
FPUF/DA-DOPO	258	267	356	372	---	---	1
FPUF/EDA-DOPO	259	279	364	380	---	---	4
FPUF/PA-DPP	219	246	357	373	---	---	0
FPUF/AA-DPP	222	247	357	372	---	---	2
FPUF/BA-DPP	267	280	352	368	---	---	2
FPUF/DA-DPP	208	232	348	364	---	---	0
FPUF/EDA-DPP	257	271	334	353	---	---	6
FPUF/PA-DEP	125	155	272	285	354	370	0
FPUF/AA-DEP	128	151	270	290	357	374	1
FPUF/BA-DEP	193	213	267	280	346	367	0
FPUF/DA-DEP	122	130	265	292	353	368	6
FPUF/EDA-DEP	241	268	333	360	---	---	3

^(a)^*T*_max_: the maximum-rate decomposition temperature.

**Table 5:** Detailed data of FPUF and flame retarded FPUF from MCC.

Samples	P-Content (wt %)	THR(kJ/g) ^(a)^	HRC (J/g K) ^(b)^	PHRR(W/g) ^(c)^	Char Residue (wt %)
FPUF	---	26.0 ± 0.9	489.0 ± 7.0	484.8 ± 12.6	1.1 ± 0.4
FPUF/PA-DOPO	2.12 ± 0.09	28.1 ± 0.9	479.0 ± 13.1	479.4 ± 11.7	1.8 ± 1.0
FPUF/AA-DOPO	2.10 ± 0.04	26.7 ± 0.8	487.7 ± 72.1	461.3 ± 21.4	1.8 ± 0.7
FPUF/BA-DOPO	1.93 ± 0.03	27.9 ± 1.5	326.3 ± 85.5	461.3 ± 21.4	0.2 ± 0.2
FPUF/DA-DOPO	2.04 ± 0.11	25.5 ± 0.8	561.7 ± 24.9	565.5 ± 20.5	0.7 ± 0.6
FPUF/EDA-DOPO	2.09 ± 0.08	23.6 ± 0.9	516. 7 ± 2.9	516.3 ± 3.0	6.5 ± 0.6
FPUF/PA-DPP	2.00 ± 0.08	26.9 ± 1.1	593.7 ± 18.3	593.7 ± 18.9	2.3 ± 2.3
FPUF/AA-DPP	2.11 ± 0.04	26.2 ± 0.8	556.7 ± 19.1	550.0 ± 13.9	0.6 ± 0.5
FPUF/BA-DPP	2.13 ± 0.01	26.8 ± 0.3	530.0 ± 4.0	529.8 ± 7.4	0.3 ± 0.2
FPUF/DA-DPP	2.08 ± 0.06	26.2 ± 2.4	581.0 ± 30.5	572.1 ± 27.7	0.8 ± 0.4
FPUF/EDA-DPP	1.98 ± 0.11	26.4 ± 0.1	387.3 ± 23.9	394.2 ± 21.7	4.7 ± 1.2
FPUF/PA-DEP	2.07 ± 0.00	26.1 ± 3.3	575.7 ± 5.7	564.2 ± 3.4	0.9 ± 0.2
FPUF/AA-DEP	2.15 ± 0.01	23.1 ± 0.8	600.0 ± 11.3	616.8 ± 9.2	0.6 ± 0.6
FPUF/BA-DEP	2.13 ± 0.00	25.8 ± 1.2	558.0 ± 14.0	546.9 ± 10.2	0.2 ± 0.2
FPUF/DA-DEP	2.00 ± 0.08	23.6 ± 1.0	659.0 ± 4.4	667.7 ± 14.3	1.8 ± 1.3
FPUF/EDA-DEP	2.07 ± 0.07	25.5 ± 0.8	490.3 ± 29.4	486.0 ± 31.9	2.1 ± 1.1

^(a)^ THR: Total heat release rate; ^(b)^ HRC: Heat release capacity (the maximum heat release rate divided by the constant heating rate); ^(c)^ PHRR: Peak heat release rate. All measurements are an average of three measurements.

## Supplementary Materials

The following are available online at http://www.mdpi.com/2073-4360/10/7/740/s1, Figure S1: NMR spectra (DMSO-d_6_) of PA-DEP, Figure S2: NMR spectra (DMSO-d_6_) of BA-DEP, Figure S3: NMR spectra (DMSO-d_6_) of AA-DEP, Figure S4: NMR spectra (DMSO-d_6_) of DA-DEP, Figure S5: NMR spectra (DMSO-d_6_) of EDA-DEP, Figure S6: NMR spectra (DMSO-d_6_) of PA-DPP, Figure S7: NMR spectra (DMSO-d_6_) of BA-DPP, Figure S8: NMR spectra (DMSO-d_6_) of AA-DPP, Figure S9: NMR spectra (DMSO-d_6_) of DA-DPP, Figure S10: NMR spectra (DMSO-d_6_) of EDA-DPP, Figure S11: NMR spectra (DMSO-d_6_) of DA-DOPO, Figure S12: NMR spectra (DMSO-d_6_) of AA-DOPO, Figure S13: TGA of the treated FPUFs in comparison with the pure FPUF (a–e), Figure S14: Heat release rate curves of the treated FPUFs in comparison with the pure FPUF (a–o), Figure S15: Total heat release of the treated FPUFs in comparison with pure FPUF, Figure S16: Peak heat release rate of the treated FPUFs in comparison with pure FPUF, Figure S17: Heat Release capacity of the treated FPUFs in comparison with pure FPUF. Figure S18: DSC curves of the solid substances (a–k).

## Abbreviations

CDCl_3_	Deuterated chloroform
DOPO	9,10-Dihydro-9-oxa-10-phosphaphenanthrene 10-oxide
FPUF	Flexible polyurethane foam
HRC	Heat release capacity
HRR	Heat release rate
MCC	Microscale combustion calorimeter
NMR	Nuclear magnetic resonance
TGA	Thermogravimetric analysis
THF	Tetrahydrofuran
THR	Total heat release rate
