# Supplementary material for: One-Pot Synthesis of P(O)-N Containing Compounds Using N-Chlorosuccinimide and Their Influence in Thermal Decomposition of PU Foams
Source: Polymers (Basel). 2018 Jul 5;10(7):740. doi: 10.3390/polym10070740 (PMC6403897; doi:10.3390/polym10070740)
Supplement: Supplementary file 1 [file polymers-10-00740-s001.pdf]

Electronic Supplementary Information

One-Pot Synthesis of P(*O*)-N Containing Compounds using *N*-Chlorosuccinimide and Their Influence in Thermal Decomposition of PU Foams

Khalifah A. Salmeia [1,*], Florence Flaig [1], Daniel Rentsch [2] and Sabyasachi Gaan [1,*]

[1] Additives and Chemistry Group, Advanced Fibers, Empa, Swiss Federal Laboratories for Materials Science and Technology, Lerchenfeldstrasse 5, 9014 St. Gallen, Switzerland.

[2] Laboratory for Functional Polymers, Empa, Swiss Federal Laboratories for Materials Science and Technology, Überlandstrasse 129, 8600 Dübendorf, Switzerland.

* Correspondence: khalifah.salmeia@empa.ch (K.A.S.); sabyasachi.gaan@empa.ch (S.G.);
Tel.: +41 58 765 7038 (K.A.S.); +41 58 765 7611 (S.G.)

Contents

**NMR peak assignments and spectra**	**2**
**Selected TGA of flame retarded FPUFs and the corresponding FR additives**	**14**
**Plot of heat release flame retarded FPUFs**	**16**
**Total heat release of flame retarded FPUFs**	**17**
**Peak heat release rate of flame retarded FPUFs**	**17**
**Heat Release capacity of flame retarded FPUFs**	**18**
**DSC curves of the solid FR additives**	**19**

^{1}H, ^{31}P{^{1}H} and ^{13}C{1H} NMR spectra were collected at ambient temperature using Bruker AV-III 400 spectrometer (Bruker Biospin AG, Fällanden, Switzerland). ^{1}H and ^{13}C chemical shifts (δ) in ppm are calibrated to residual solvent peaks at 2.49 and 39.5 ppm, respectively. The ^{31}P chemical shifts were referenced to an external sample with neat H_3PO_4 at 0.0 ppm. For ^{13}C NMR data multiplicities *s* = quaternary carbon, *d* = CH, *t* = CH_2, and *q* = CH_3 are shown and ^{31}P,^{13}C, ^{1}H,^{1}H and ^{1}H,^{31}P coupling constants are reported in Hz.

Diethyl propylphosphoramidate (PA-DEP): after removing the reaction solvent, the product was extracted with diethyl ether from the reaction residue.

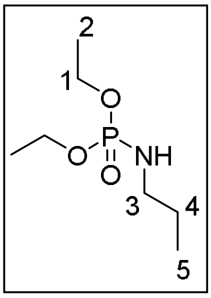

^{1}H NMR (400.2 MHz, DMSO-d_6) δ (ppm): 4.78 (m, 1H, NH); 3.87 (td, J_{HH} = 7.3, J_{HP} = 7.3, 4H, H-1); 2.67 (qd, J_{HH} = 6.9, J_{HP} = 11.0, 2H, H-3); 1.38 (m, 2H, H-4); 1.19 (t, J_{HH} = 7.3, 6H, H-2); 0.82 (t, J_{HH} = 7.4, 3H, H-5). ^{13}C NMR (100.6 MHz, DMSO-d_6) δ (ppm): 61.0 (td, J_{CP} = 5.2, C-1); 42.6 (t, C-3); 24.5 (td, J_{CP} = 5.7, C-4); 16.1 (qd, J_{CP} = 6.8, C-2); 11.2 (q, C-5). ^{31}P{^{1}H} NMR (162.0 MHz, DMSO-d_6) δ (ppm): 10.0. *Anal.* Calc. for [$C_7H_{18}NO_3P$]: P, 15.87. Found: P, 15.52%.

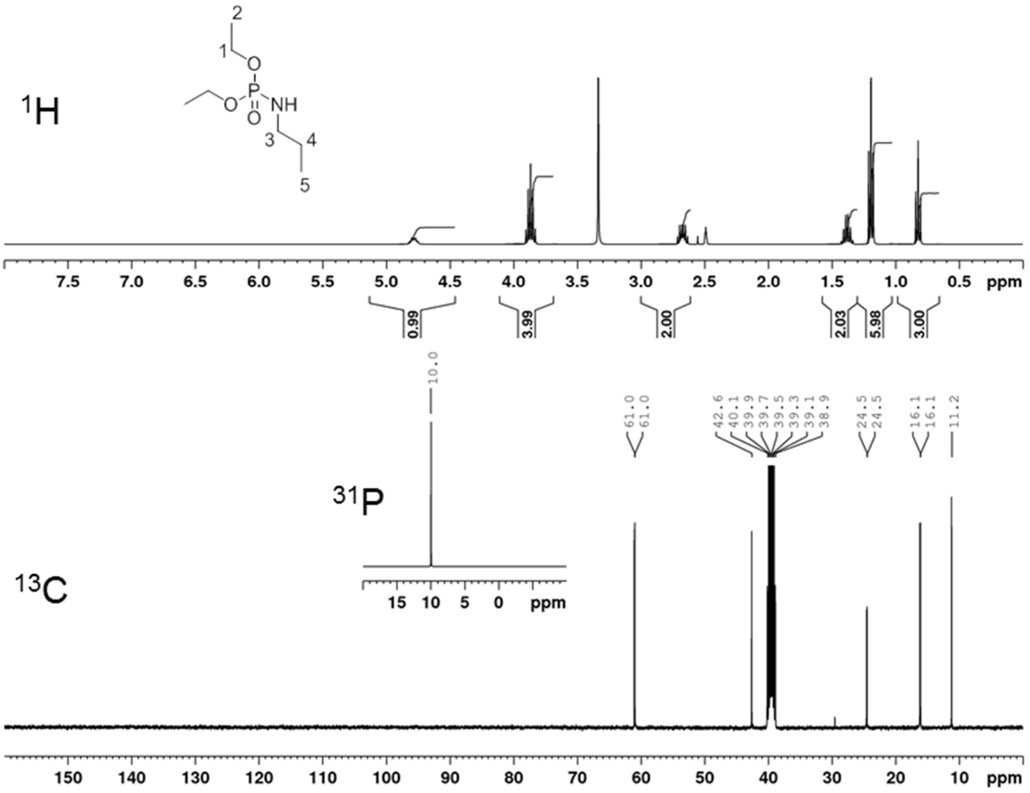

Figure S1: NMR Spectra (DMSO-d_6) of PA-DEP

Diethyl benzylphosphoramidate (BA-DEP): after removing the reaction solvent, the product was extracted with diethyl ether from the reaction residue. After filtration, the solvent was removed, affording the product as pale yellow oil.

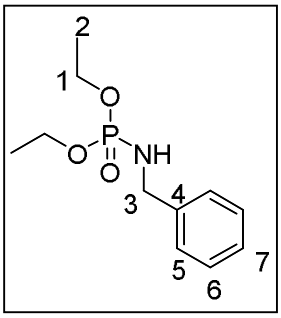

^{1}H NMR (400.2 MHz, DMSO-d$_6$) δ (ppm): 7.25-7.35 (m, 4H, H-5&6); 7.22 (m, 1H, H-7); 5.42 (td, J_{HH} = 7.3, J_{HP} = 12.1, 1H, NH); 3.94 (dd, J_{HH} = 7.4, J_{HP} = 12.0, 2H, H-3); 3.85 (m, 4H, H-1); 1.15 (td, J_{HH} = 7.1, J_{HP} = 0.7, 6H, H-2). ^{13}C NMR (100.6 MHz, DMSO-d$_6$) δ (ppm): 140.9 (sd, J_{CP} = 5.1, C-4); 128.1 (d, C-6); 127.1 (d, C-5); 126.7 (d, C-7); 61.2 (td, J_{CP} = 5.3, C-1); 44.3 (t, C-3); 16.0 (qd, J_{CP} = 6.8, C-2). ^{31}P{^{1}H} NMR (162.0 MHz, DMSO-d$_6$) δ (ppm): 9.7. *Anal.* Calc. for [$C_{11}H_{18}NO_3P$]: P, 12.73. Found: P, 12.01%.

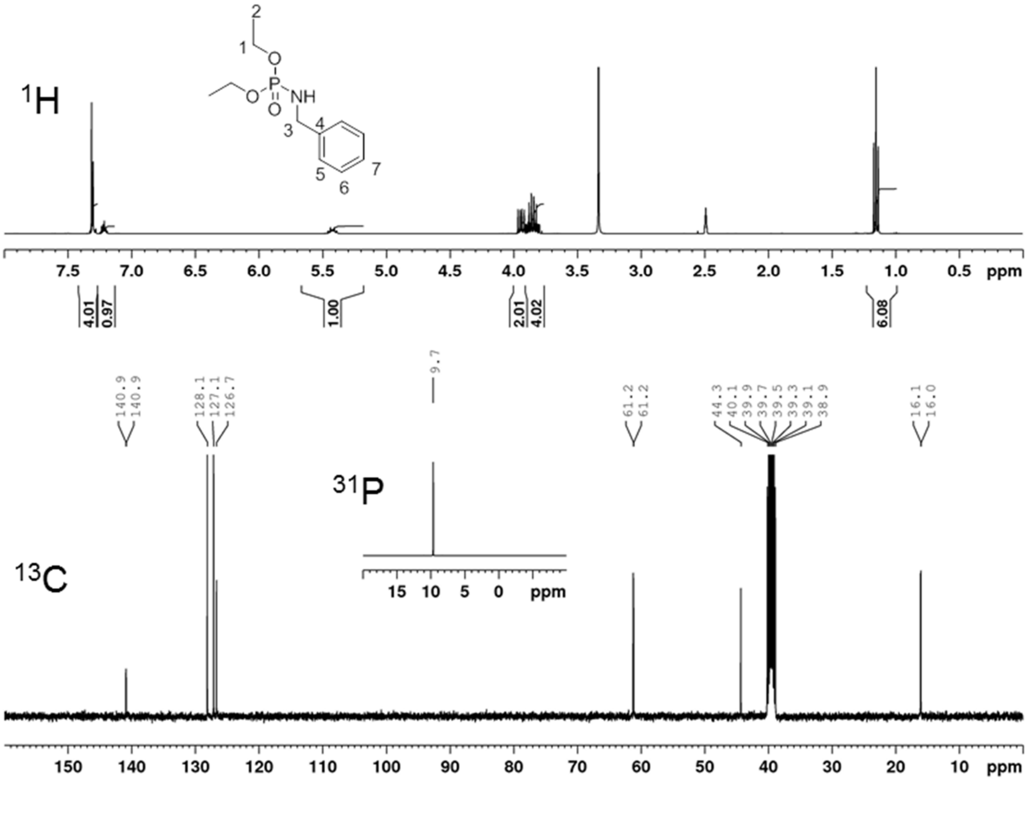

Figure S2: NMR Spectra (DMSO-d$_6$) of BA-DEP.

Diethyl allylphosphoramidate (AA-DEP): after removing the reaction solvent, the product was purified by column chromatography using ethyl acetate as eluent.

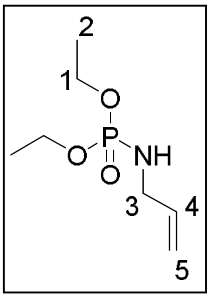

^{1}H NMR (400.2 MHz, DMSO-d$_6$) δ (ppm): 5.80 (m, 1H, H-4); 5.0-5.2 (m, 3H, H-5, NH); 3.88 (td, J_{HH} = 7.3, J_{HP} = 7.3, 4H, H-1); 3.36 (m, 2H, H-3); 1.19 (td, J_{HH} = 7.3, J_{HP} = 0.6, 6H, H-2). ^{13}C NMR (100.6 MHz, DMSO-d$_6$) δ (ppm): 137.3 (dd, J_{CP} = 5.5, C-4); 114.5 (t, C-5); 61.1 (td, J_{CP} = 5.3, C-1); 43.1 (t, C-3); 16.1 (qd, J_{CP} = 6.8, C-2). ^{31}P{^{1}H} NMR (162.0 MHz, DMSO-d$_6$) δ (ppm): 9.7. *Anal.* Calc. for [C7H16NO3P]: P, 16.03. Found: P, 14.38%.

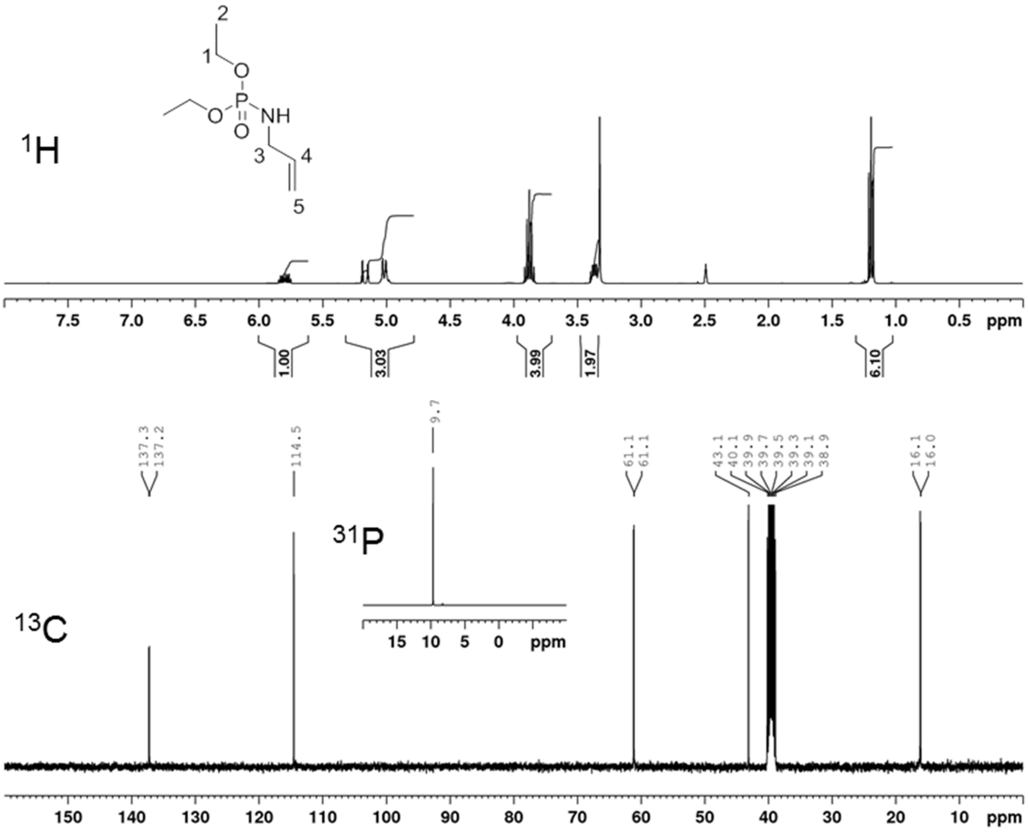

Figure S3: NMR Spectra (DMSO-d$_6$) of AA-DEP.

Diethyl diethylphosphoramidate (DA-DEP): after removing the reaction solvent, the product was extracted with diethyl ether from the reaction residue. After filtration, the solvent was removed, affording the product as pale yellow oil.

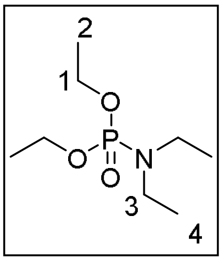

^{1}H NMR (400.2 MHz, DMSO-d$_6$) δ (ppm): 3.85 (m, 4H, H-1); 2.96 (qd, J_{HH} = 7.1, J_{HP} = 11.5, 4H, H-3); 1.20 (t, J_{HH} = 7.1, 6H, H-2); 1.01 (t, J_{HH} = 7.1, 6H, H-4). ^{13}C NMR (100.6 MHz, DMSO-d$_6$) δ (ppm): 61.5 (td, J_{CP} = 5.2, C-1); 39.6 (t, C-3); 16.5 (qd, J_{CP} = 6.8, C-2); 14.6 (td, J_{CP} = 1.6, C-4). ^{31}P{^{1}H} NMR (162.0 MHz, DMSO-d$_6$) δ (ppm): 10.2. *Anal.* Calc. for [$C_8H_{20}NO_3P$]: P, 14.80. Found: P, 14.43%.

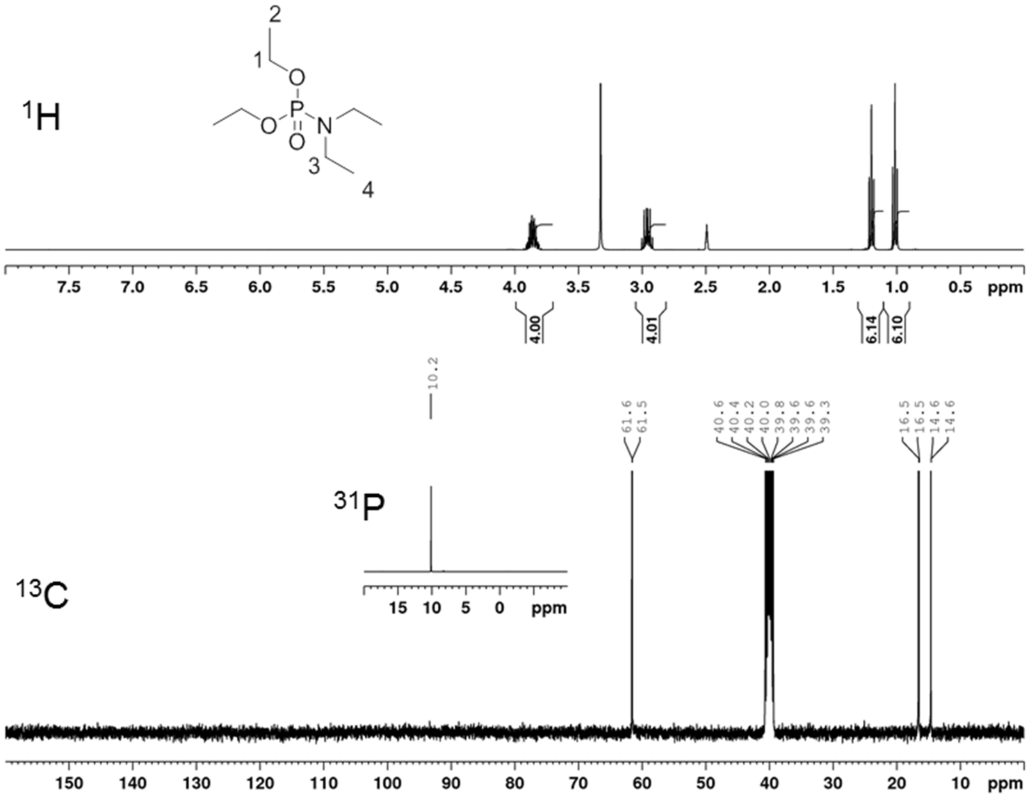

Figure S4: NMR Spectra (DMSO-d$_6$) of DA-DEP.

Tetraethyl ethane-1,2-diylbis(phosphoramidate) (EDA-DEP): after removing the reaction solvent, the product was extracted with warm THF from the reaction residue. After filtration, the solvent was removed, affording the product as an off-white solid.

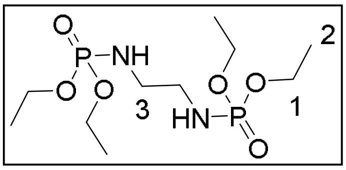

^{1}H NMR (400.2 MHz, DMSO-d$_6$) δ (ppm): 4.83 (m, 2H, NH); 3.87 (qd, J_{HH} = 7.1, J_{HP} = 7.3, 8H, H-1); 2.76 (m, 4H, H-3); 1.19 (td, J_{HH} = 7.1, J_{HP} = 0.6, 12H, H-2). ^{13}C NMR (100.6 MHz, DMSO-d$_6$) δ (ppm): 61.2 (td, J_{CP} = 5.3, C-1); 42.2 (td, J_{CP} = 6.0, C-3); 16.1 (qd, J_{CP} = 6.7, C-2). ^{31}P{^{1}H} NMR (162.0 MHz, DMSO-d$_6$) δ (ppm): 9.7. *Anal.* Calc. for [$C_{10}H_{26}N_2O_6P_2$]: P, 18.64. Found: P, 17.79%.

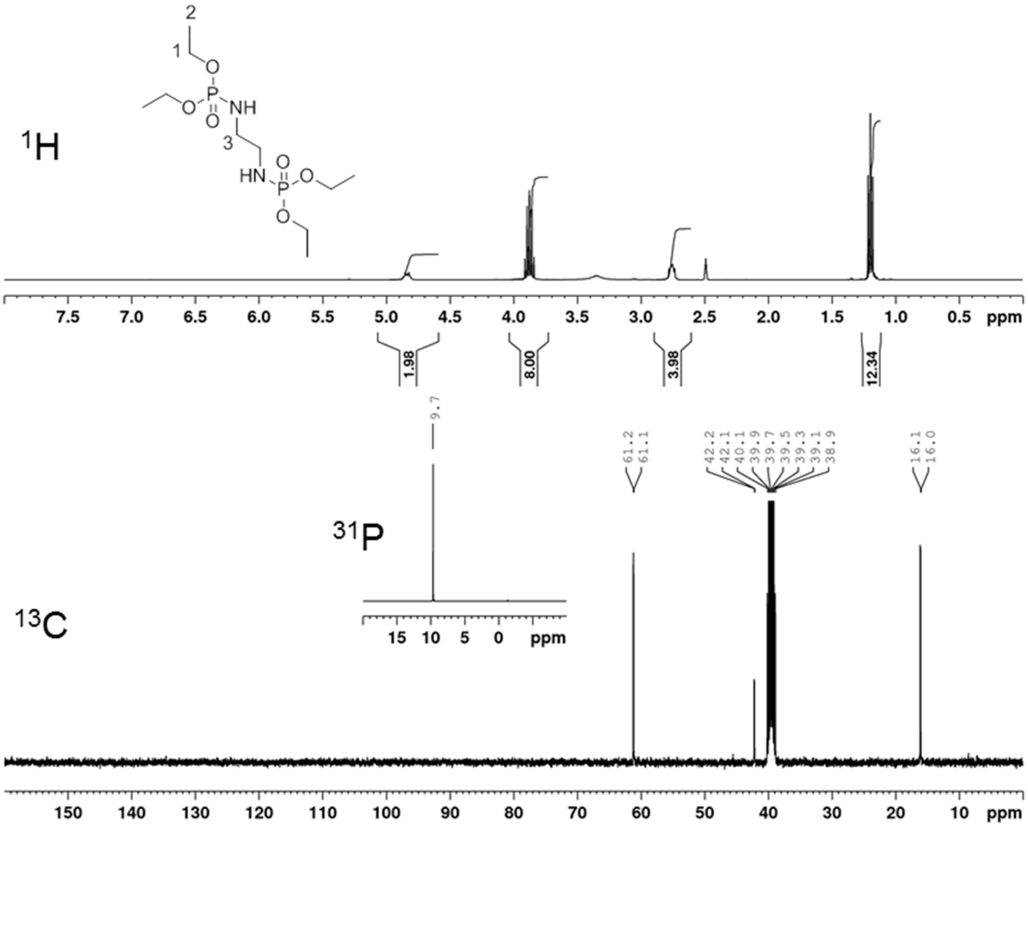

Figure S5: NMR Spectra (DMSO-d$_6$) of EDA-DEP.

Diphenyl propylphosphoramidate (PA-DPP): the product was purified by Column chromatography using hexane as eluent, affording an off-white product

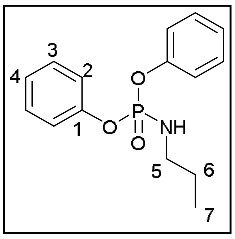

^{1}H NMR (400.2 MHz, DMSO-d$_6$) δ (ppm): 7.38 (m, 4H, H-3); 7.15-7.25 (m, 6H, H-2, 4); 5.78 (td, J_{HH} = 6.7, J_{HP} = 13.7, 1H, NH); 2.85 (m, 2H, H-5); 1.35 (m, 2H, H-6); 0.76 (t, J_{HH} = 7.4, 3H, H-7). ^{13}C NMR (100.6 MHz, DMSO-d$_6$) δ (ppm): 150.7 (sd, J_{CP} = 6.4, C-1); 129.8 (d, C-3); 124.7 (dd, J_{CP} = 1.1, C-4); 120.1 (dd, J_{CP} = 4.9, C-2); 42.9 (t, C-5); 24.2 (td, J_{CP} = 5.9, C-6); 11.0 (q, C-7). ^{31}P{^{1}H} NMR (162.0 MHz, DMSO-d$_6$) δ (ppm): 0.9. *Anal.* Calc. for [$C_{15}H_{18}NO_3P$]: P, 10.63. Found: P, 10.32%.

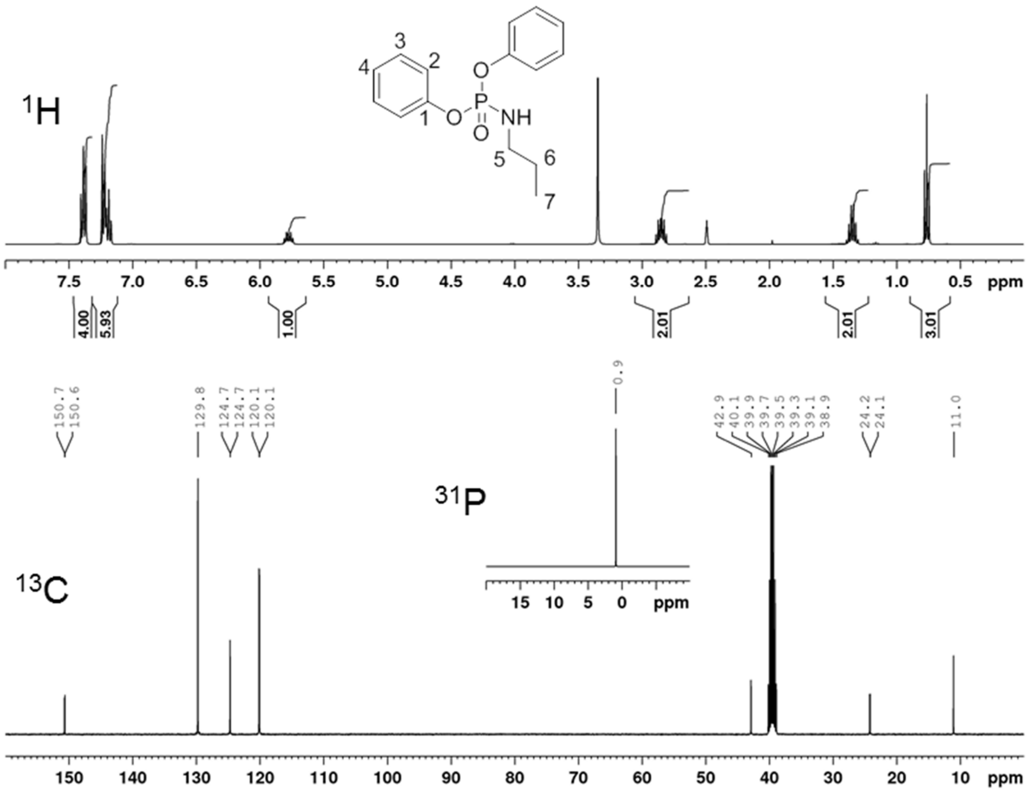

Figure S6: NMR Spectra (DMSO-d$_6$) of PA-DPP.

Diphenyl benzylphosphoramidate (BA-DPP): after removing the volatiles, the residue was stirred in water, affording the product as an off-white solid.

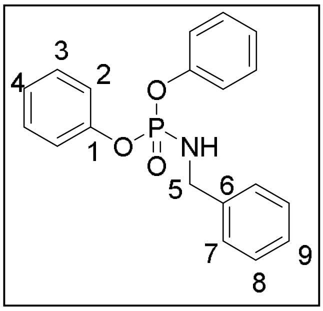

^{1}H NMR (400.2 MHz, DMSO-d$_6$) δ (ppm): 7.37 (m, 4H, H-3); 7.2-7.3 (m, 11H, H-2, 4, 7-9); 6.37 (td, J_{HH} = 7.0, J_{HP} = 14.0, 1H, NH); 4.13 (dd, J_{HH} = 7.0, J_{HP} = 12.8, 2H, H-5). ^{13}C NMR (100.6 MHz, DMSO-d$_6$) δ (ppm): 150.6 (sd, J_{CP} = 6.6, C-1); 139.9 (sd, J_{CP} = 5.4, C-6); 129.8 (d, C-3); 128.1 (d, C-8); 127.2 (d, C-7); 126.9 (d, C-9); 124.8 (dd, J_{CP} = 1.1, C-4); 120.2 (dd, J_{CP} = 4.8, C-2); 44.6 (t, C-5). ^{31}P{^{1}H} NMR (162.0 MHz, DMSO-d$_6$) δ (ppm): 0.7. *Anal.* Calc. for [$C_{19}H_{18}NO_3P$]: P, 9.13. Found: P, 8.39%.

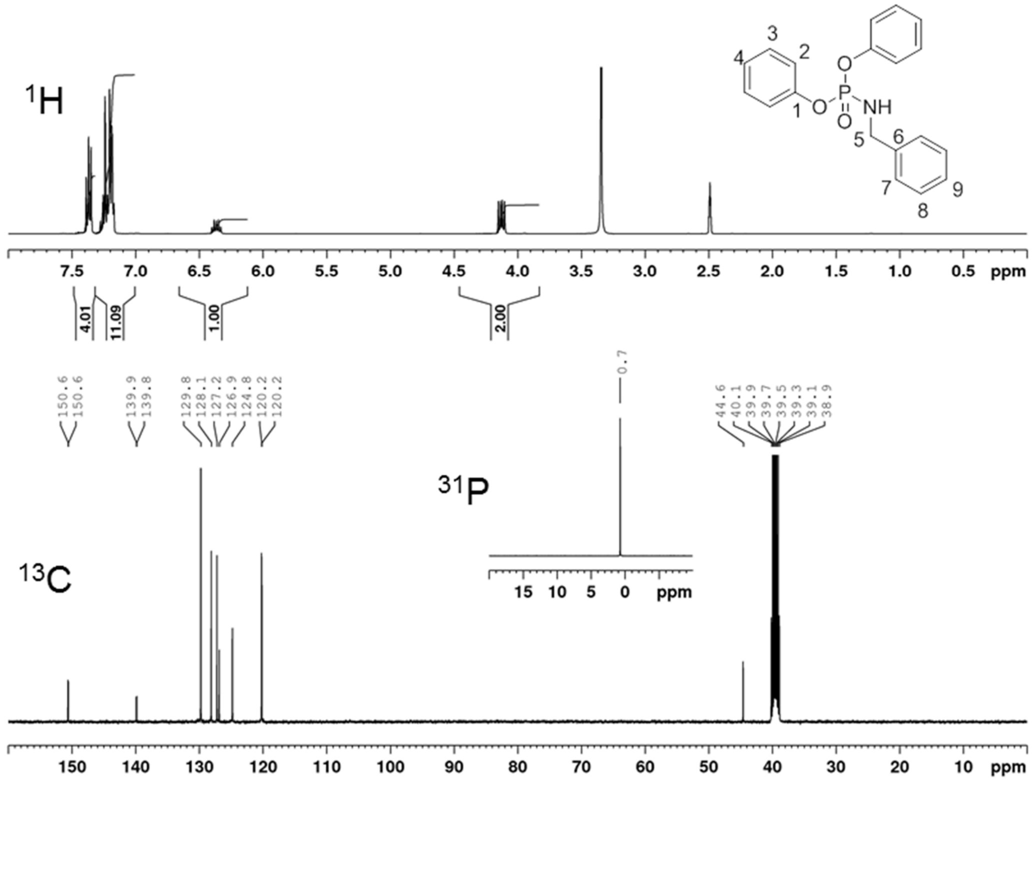

Figure S7: NMR Spectra (DMSO-d$_6$) of BA-DPP.

Diphenyl allylphosphoramidate (AA-DPP): after removing the volatiles, the residue was stirred in water, affording the product as a white solid.

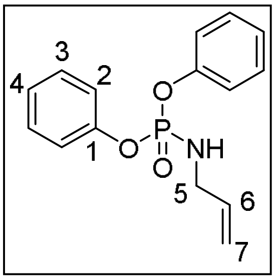

^{1}H NMR (400.2 MHz, DMSO-d$_6$) δ (ppm): 7.39 (m, 4H, H-3); 7.15-7.25 (m, 6H, H-2, 4); 6.00 (td, J_{HH} = 6.8, J_{HP} = 13.8, 1H, NH); 5.72 (m, 1H, H-6); 5.13+4.99 (m, 2H, H-7); 3.55 (m, 2H, H-5). ^{13}C NMR (100.6 MHz, DMSO-d$_6$) δ (ppm): 150.6 (sd, J_{CP} = 6.5, C-1); 136.2 (dd, J_{CP} = 5.6, C-6); 129.8 (d, C-3); 124.8 (dd, J_{CP} = 1.1, C-4); 120.2 (dd, J_{CP} = 4.8, C-2); 115.2 (t, C-7); 43.3 (t, C-5). ^{31}P{^{1}H} NMR (162.0 MHz, DMSO-d$_6$) δ (ppm): 0.7. *Anal.* Calc. for [$C_{15}H_{16}NO_3P$]: P, 10.71. Found: P, 10.26%.

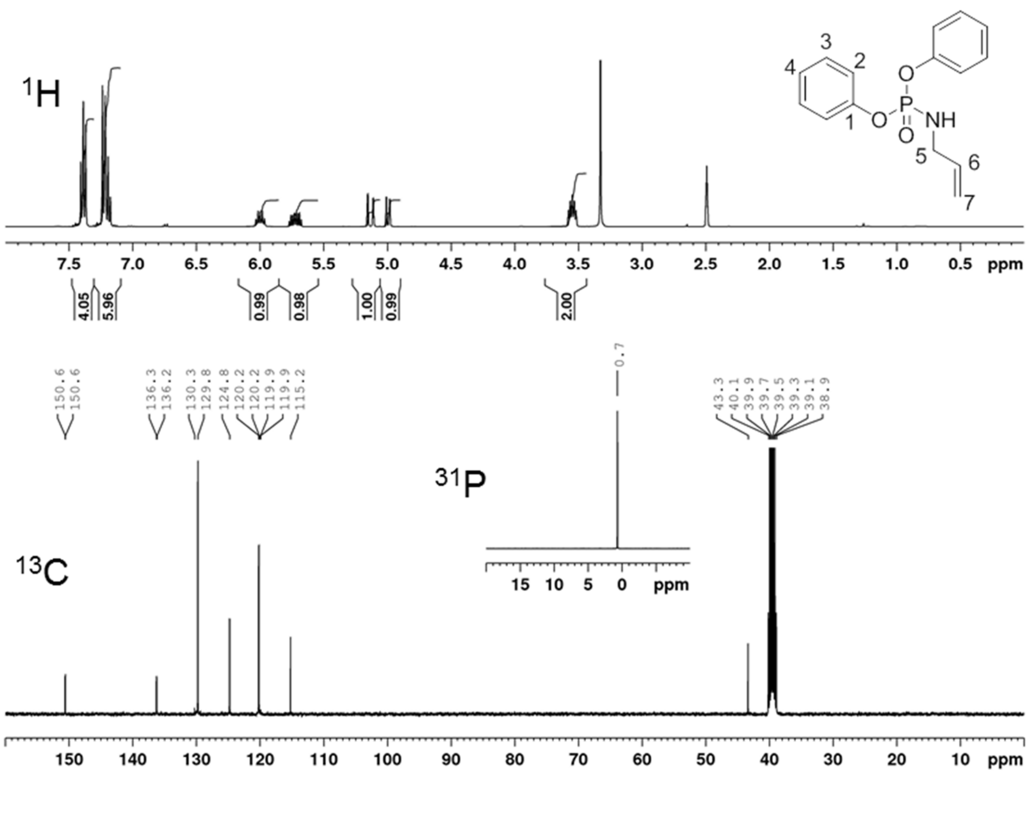

Figure S8: NMR Spectra (DMSO-d$_6$) of AA-DPP.

Diphenyl diethylphosphoramidate (DA-DPP): the product was purified by column chromatography using heptane as an eluent, affording colourless oil which was solidified with time.

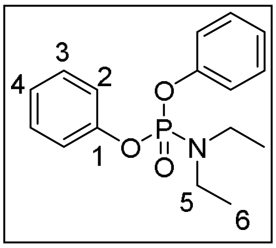

^{1}H NMR (400.2 MHz, DMSO-d$_6$) δ (ppm): 7.39 (m, 4H, H-3); 7.15-7.25 (m, 6H, H-2, 4); 3.12 (qd, J_{HH} = 7.1, J_{HP} = 12.2, 4H, H-5); 0.93 (t, J_{HH} = 7.1, 6H, H-6). ^{13}C NMR (100.6 MHz, DMSO-d$_6$) δ (ppm): 150.4 (sd, J_{CP} = 6.4, C-1); 129.8 (d, C-3); 124.9 (dd, J_{CP} = 1.0, C-4); 120.0 (dd, J_{CP} = 5.0, C-2); 39.2 (t, C-5); 13.6 (qd, J_{CP} = 1.8, C-6). ^{31}P{^{1}H} NMR (162.0 MHz, DMSO-d$_6$) δ (ppm): 1.0. *Anal.* Calc. for [$C_{16}H_{20}NO_3P$]: P, 10.14. Found: P, 9.68%.

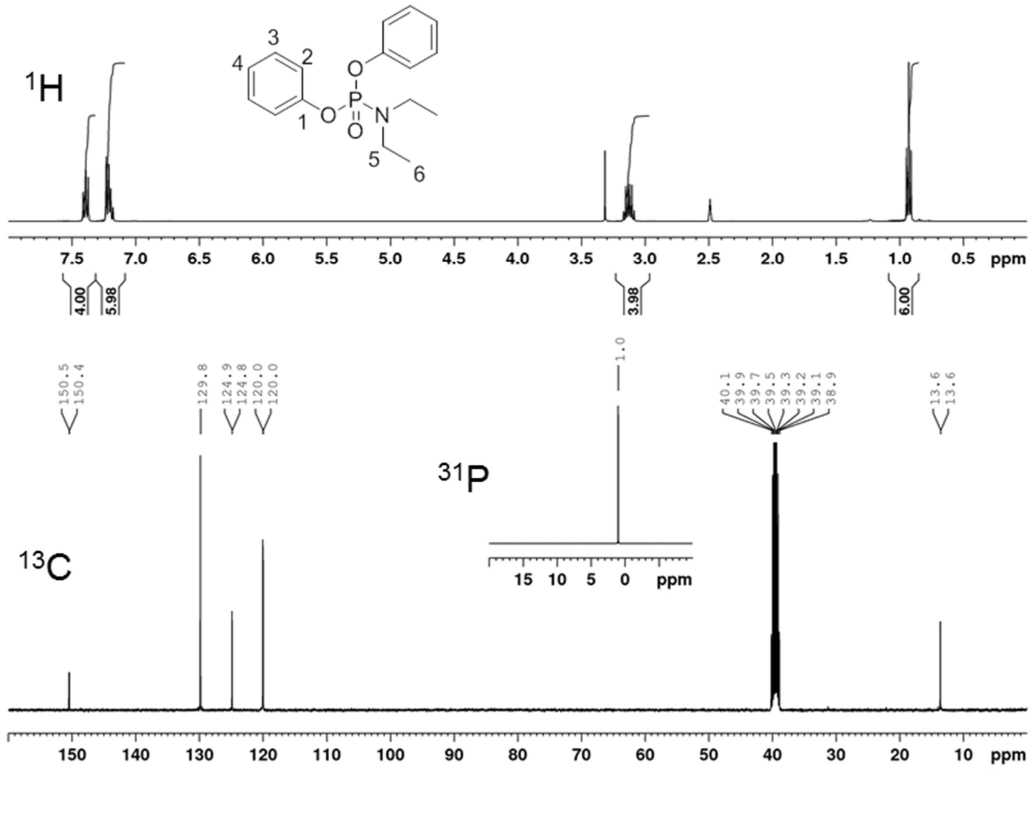

Figure S9: NMR Spectra (DMSO-d$_6$) of DA-DPP.

Tetraphenyl ethane-1,2-diylbis(phosphoramidate) (EDA-DPP): after removing the volatiles, the residue was stirred in water, affording the product as white solid.

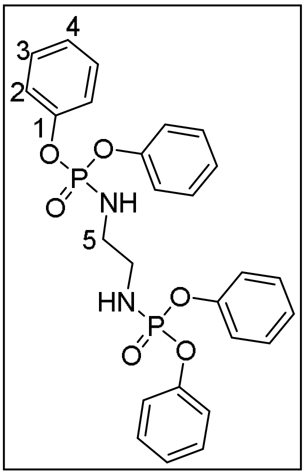

^{1}H NMR (400.2 MHz, DMSO-d$_6$) δ (ppm): 7.38 (m, 8H, H-3); 7.15-7.25 (m, 12H, H-2, 4); 5.84 (td, J_{HH} = 6.0, J_{HP} = 13.8, 2H, NH); 2.86 (m, 4H, H-5). ^{13}C NMR (100.6 MHz, DMSO-d$_6$) δ (ppm): 150.5 (sd, J_{CP} = 6.4, C-1); 129.8 (d, C-3); 124.9 (dd, J_{CP} = 1.0, C-4); 120.1 (dd, J_{CP} = 4.8, C-2); 42.0 (td, J_{CP} = 6.1, C-5). ^{31}P{^{1}H} NMR (162.0 MHz, DMSO-d$_6$) δ (ppm): 0.5. *Anal.* Calc. for [$C_{26}H_{26}N_2O_6P_2$]: P, 11.81. Found: P, 11.35%.

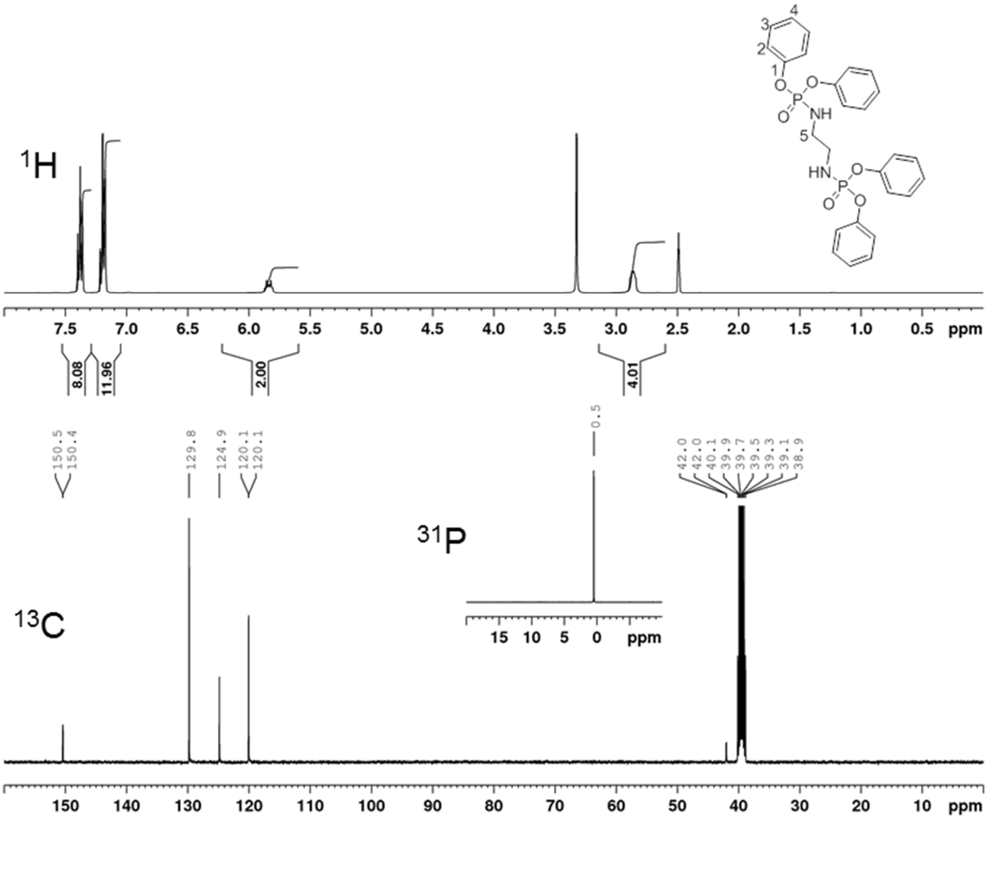

Figure S10: NMR Spectra (DMSO-d$_6$) of EDA-DPP

6-(diethylamino)dibenzo[c,e][1,2]oxaphosphinine 6-oxide (DA-DOPO): the reaction solvent was removed completely to yield viscous oil which was further stirred in water and then extracted with diethyl ether (150mL), washed with water (3x100mL) and dried over anhydrous Na_2SO_4. After filtration, the solvent was completely removed in vacuum affording the product as red solid

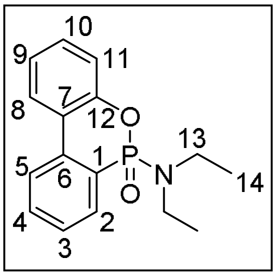

^{1}H NMR (400.2 MHz, DMSO-d$_6$) δ (ppm): 8.22 (dd, J_{HH} = 8.1, J_{HP} = 6.0, 1H, H-5); 8.18 (dd, J_{HH} = 8.1, 1.5, 1H, H-8); 7.7-7.8 (m, 2H, H-2, 4); 7.59 (m, 1H, H-3); 7.43 (m, 1H, H-10); 7.30 (m, 1H, H-9); 7.26 (dd, J_{HH} = 8.1, 1.2, 1H, H-11); 3.04 (m, 4H, H-13); 1.06 (t, J_{HH} = 7.1, 6H, H-14). ^{13}C NMR (100.6 MHz, DMSO-d$_6$) δ (ppm): 149.7 (sd, J_{CP} = 7.6, C-12); 136.5 (sd, J_{CP} = 6.9, C-6); 132.9 (dd, J_{CP} = 2.5, C-4); 130.5 (d, C-10); 129.3 (dd, J_{CP} = 9.6, C-2); 128.6 (dd, J_{CP} = 14.4, C-3); 125.4 (dd, J_{CP} = 1.0, C-8); 124.6 (sd, J_{CP} = 164.6, C-1); 124.4 (dd, J_{CP} = 0.8, C-9); 124.1 (dd, J_{CP} = 10.9, C-5); 121.4 (sd, J_{CP} = 11.5, C-7); 120.1 (dd, J_{CP} = 6.1, C-11); 38.5 (td, J_{CP} = 5.0, C-13); 14.1 (qd, J_{CP} = 2.1, C-14). ^{31}P{^{1}H} NMR (162.0 MHz, DMSO-d$_6$) δ (ppm): 15.9. *Anal.* Calc. for [$C_{16}H_{18}NO_2P$]: P, 10.78. Found: P, 10.20%.

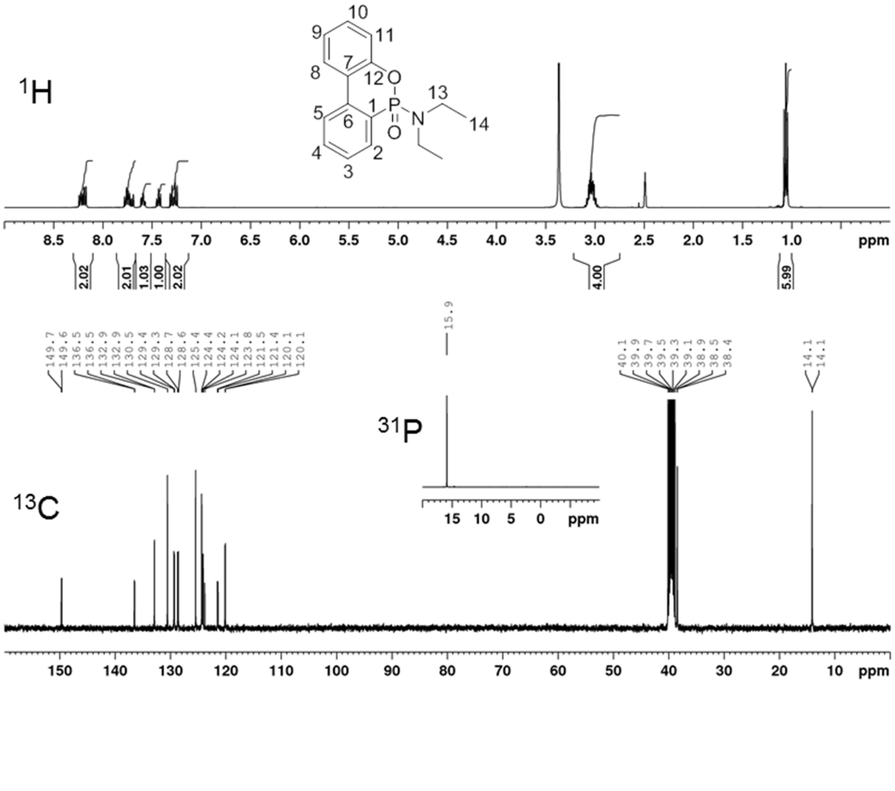

Figure S11: NMR Spectra (DMSO-d$_6$) of DA-DOPO.

6-(allylamino)dibenzo[c,e][1,2]oxaphosphinine 6-oxide (AA-DOPO)

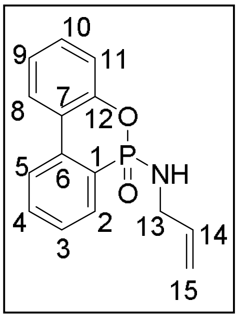

^{1}H NMR (400.2 MHz, DMSO-d_6) δ (ppm): ^{1}H NMR (400.2 MHz, DMSO-d_6) δ (ppm): 8.18 (dd, J_{HH} = 8.1, J_{HP} = 5.7, 1H, H-5); 8.16 (dd, J_{HH} = 7.9, 1.6, 1H, H-8); 7.81 (ddd, J_{HH} = 7.6, 1.1, J_{HP} = 14.0, 1H, H-2);7.74 (m, 1H, H-4); 7.58 (m, 1H, H-3); 7.43 (m, 1H, H-10); 7.29 (m, 1H, H-9); 7.25 (dd, J_{HH} = 8.1, 1.0, 1H, H-11); 5.90 (td, J_{HH} = 6.7, J_{HP} = 12.1, 1H, NH); 5.81 (m, 1H, H-14); 5.18+5.03 (m, 2H, H-15); 3.45 (m, 2H, H-13). ^{13}C NMR (100.6 MHz, DMSO-d_6) δ (ppm): 149.5 (sd, J_{CP} = 7.2, C-12); 136.7 (dd, J_{CP} = 5.4, C-14); 136.0 (sd, J_{CP} = 6.9, C-6);132.7 (dd, J_{CP} = 2.3, C-4); 130.4 (d, C-10); 129.5 (dd, J_{CP} = 9.6, C-2); 128.3 (dd, J_{CP} = 14.2, C-3); 125.6 (sd, J_{CP} = 162.3, C-1); 125.4 (dd, J_{CP} = 0.9, C-8); 124.2 (d, C-9); 124.1 (dd, J_{CP} = 10.8, C-5); 122.0 (sd, J_{CP} = 11.6, C-7); 120.1 (dd, J_{CP} = 6.0, C-11); 115.0 (t, C-15); 42.7 (t, C-13)). ^{31}P{^{1}H} NMR (162.0 MHz, DMSO-d_6) δ (ppm): 14.7. *Anal.* Calc. for [$C_{15}H_{14}NO_2P$]: P, 11.42. Found: P, 11.01%.

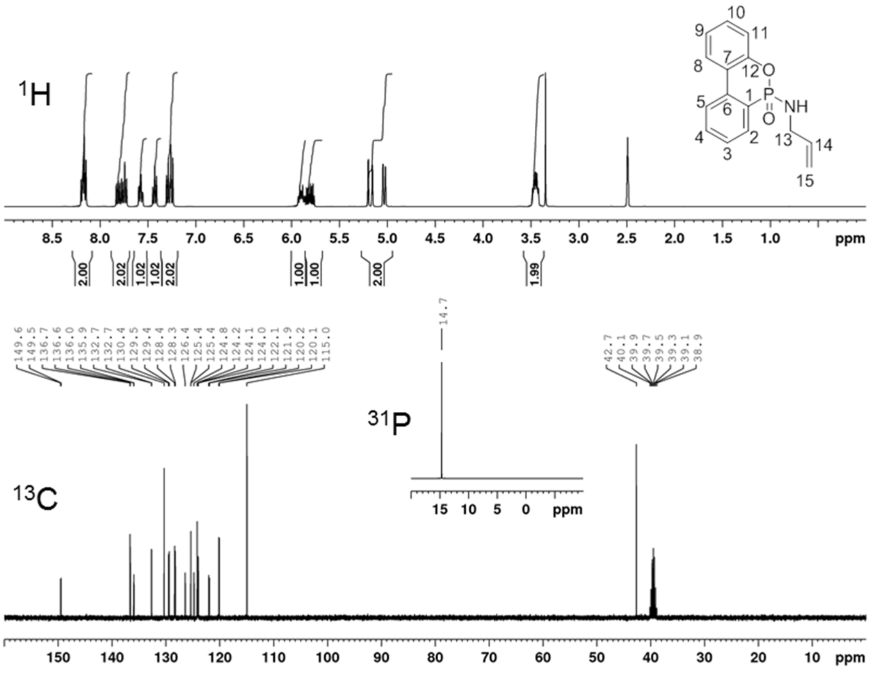

Figure S12: NMR Spectra (DMSO-d_6) of AA-DOPO.

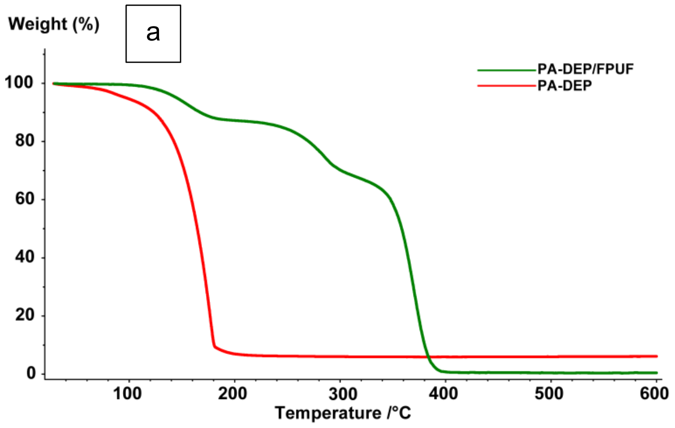

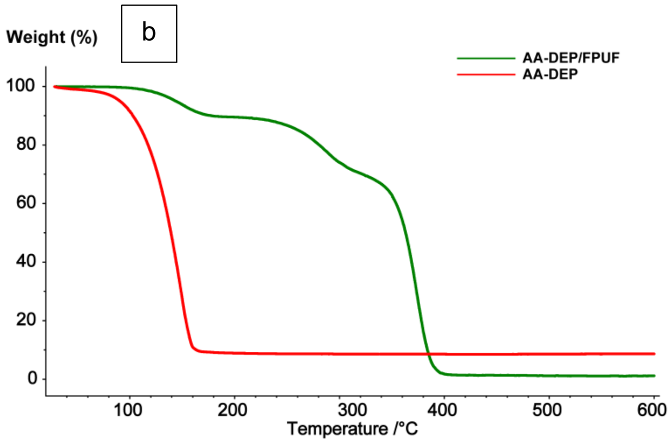

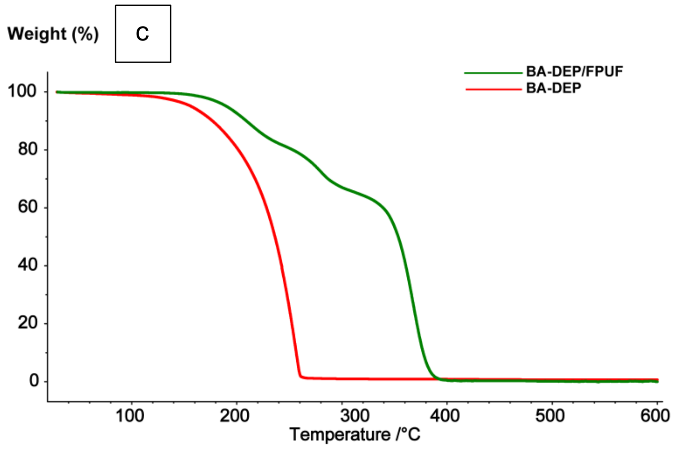

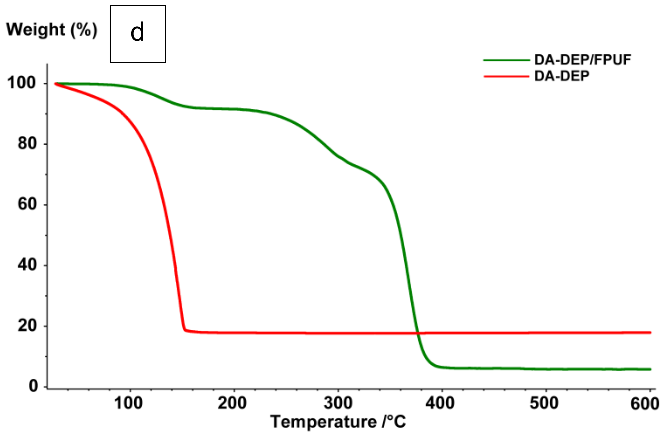

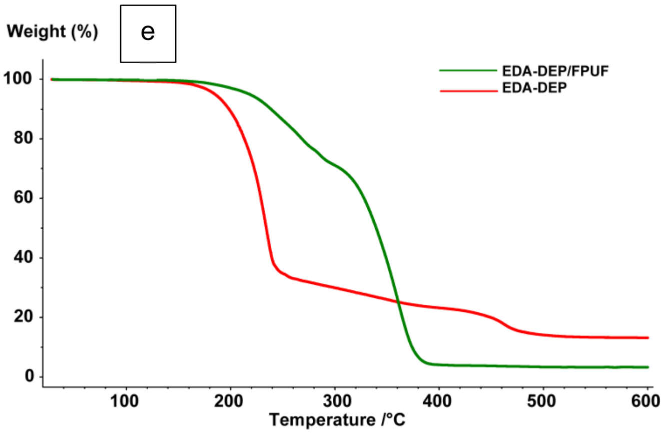

Figure S13: TGA of the treated FPUFs in comparison with the pure FPUF (a-e)

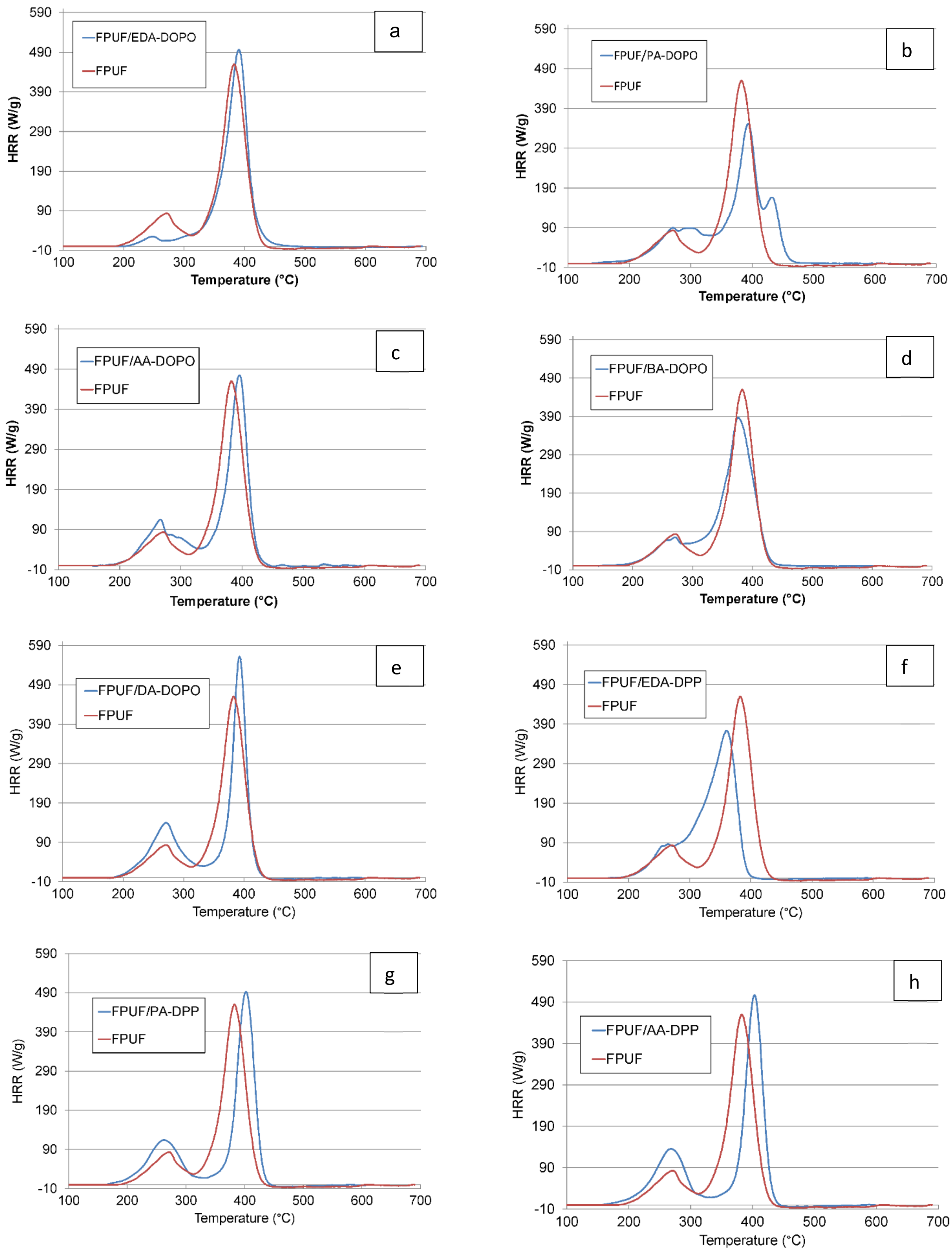
a
FPUF/EDA-DOPO
FPUF
b
FPUF/PA-DOPO
FPUF
c
FPUF/AA-DOPO
FPUF
d
FPUF/BA-DOPO
FPUF
e
FPUF/DA-DOPO
FPUF
f
FPUF/EDA-DPP
FPUF
g
FPUF/PA-DPP
FPUF
h
FPUF/AA-DPP
FPUF
HRR (W/g)
Temperature (°C)
590
490
390
290
190
90
-10
100
200
300
400
500
600
700

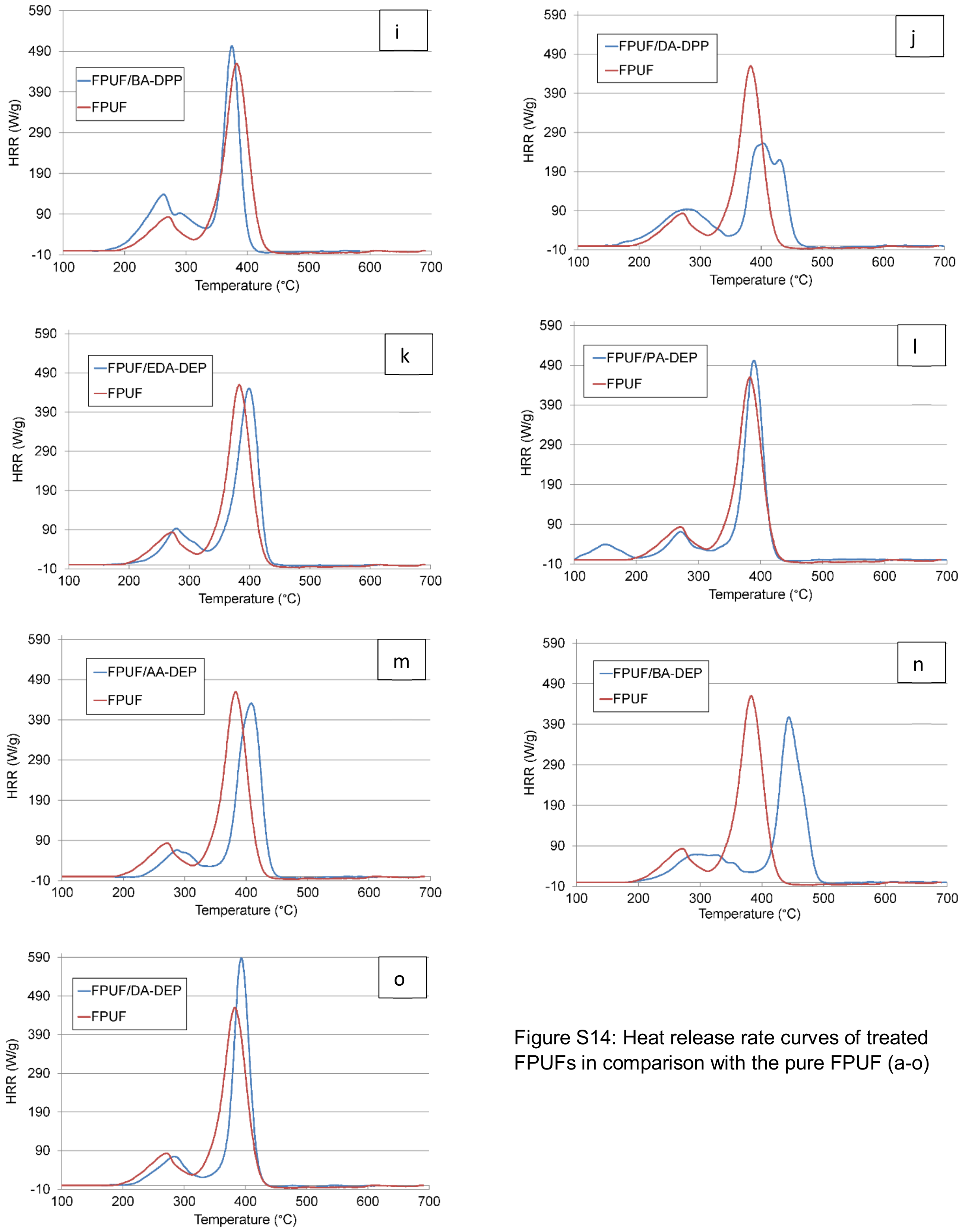

Figure S14: Heat release rate curves of treated FPUFs in comparison with the pure FPUF (a-o)

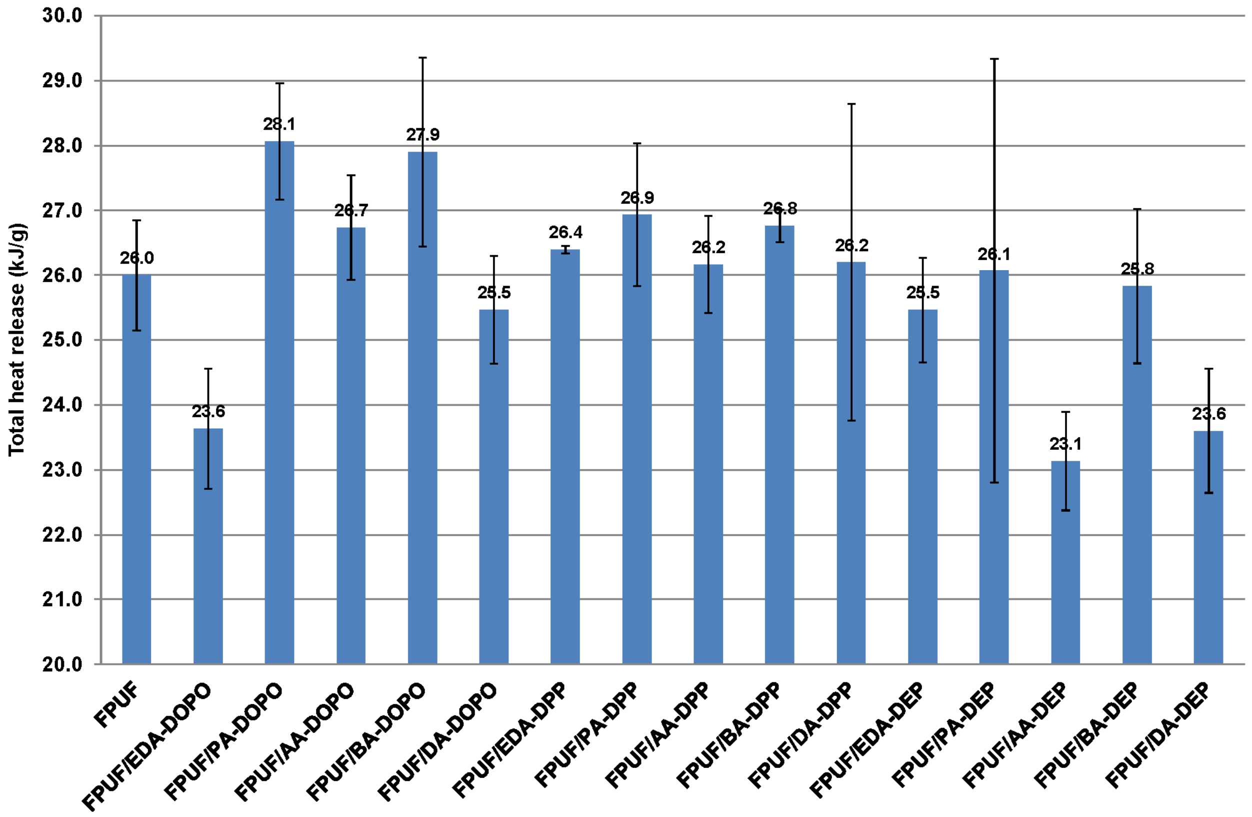

Figure S13: Total heat release of the treated FPUFs in comparison with pure FPUF

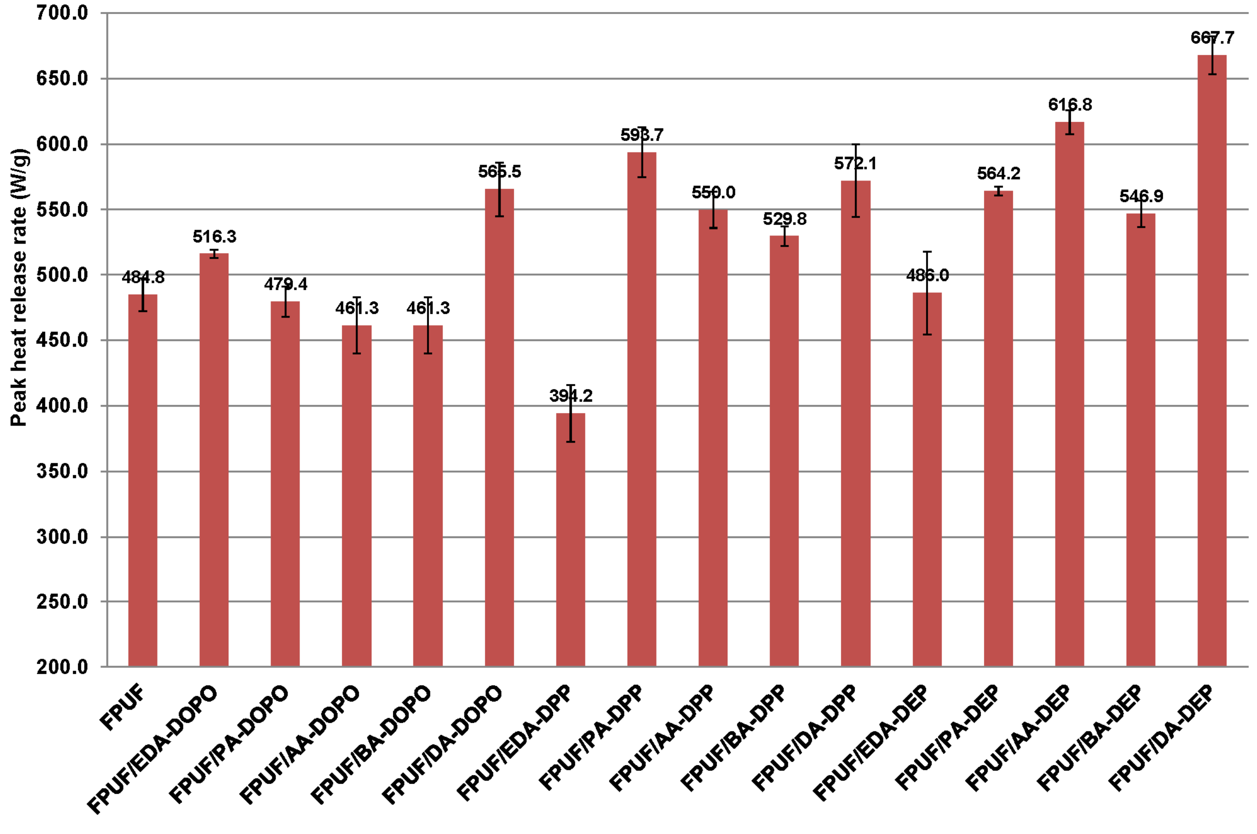

Figure S16: Peak heat release rate of the treated FPUFs in comparison with pure FPUF

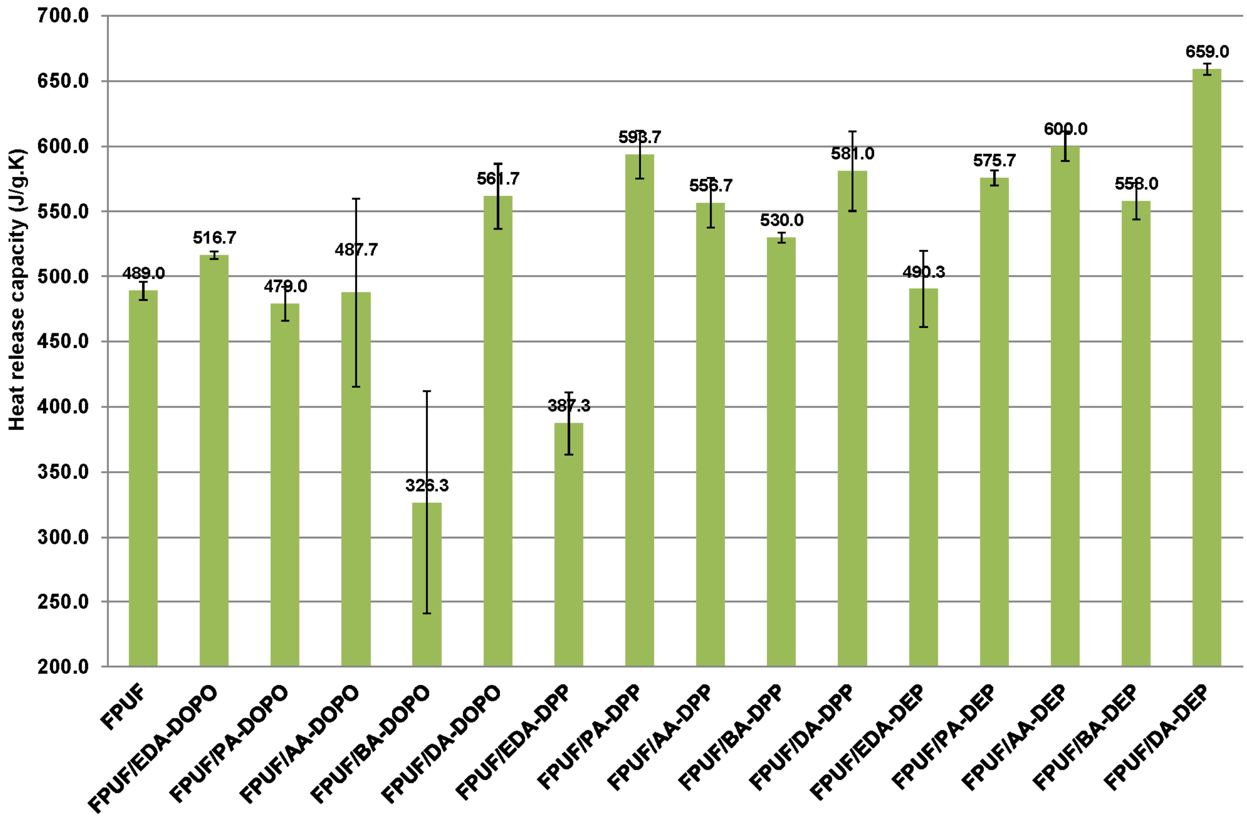

Figure S17: Heat Release capacity of the treated FPUFs in comparison with pure FPUF

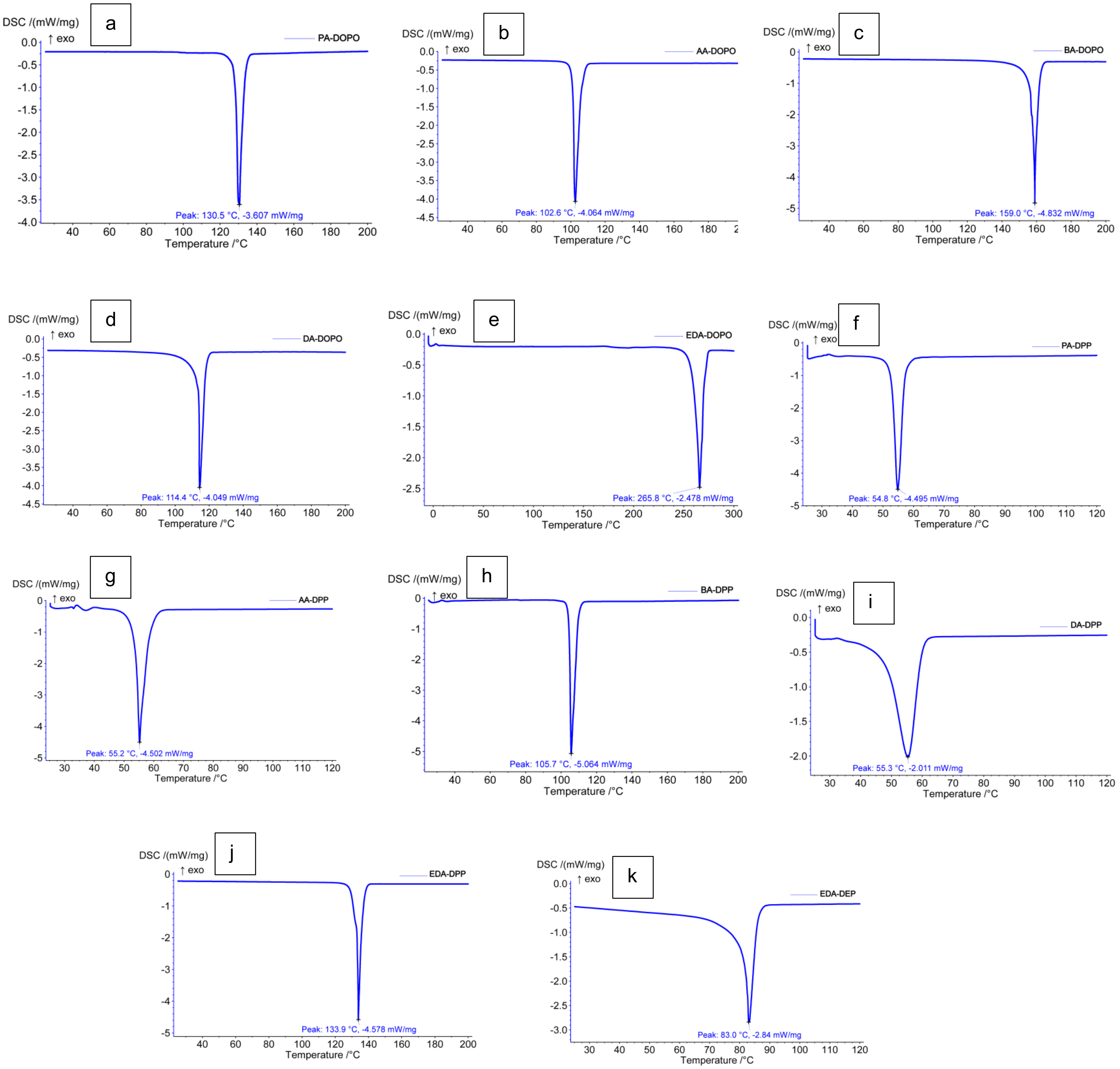

Figure S18: DSC curves of the solid substances (a-k)
